# Targeting CXCR6 Disrupts β-Catenin Signaling and Enhances Sorafenib Response in Hepatocellular Carcinoma

**DOI:** 10.3390/cancers17233818

**Published:** 2025-11-28

**Authors:** Morgan Reeves, Anastasia Chambers, Abhishek Shrestha, Sergio Duarte, Ali Zarrinpar, Siobhan Malany, Satyamaheshwar Peddibhotla

**Affiliations:** 1 Department of Cellular and Systems Pharmacology, University of Florida College of Pharmacy, 1225 Center Drive, Gainesville, FL 32610, USA; morgan.reeves@ufl.edu (M.R.); smalany@ufl.edu (S.M.); 2 Department of Surgery, University of Florida College of Medicine, 1600 SW Archer Rd, Gainesville, FL 32610, USA; agchambers@ufl.edu (A.C.); abhishek.shrestha@surgery.ufl.edu (A.S.); sergio.duarte@surgery.ufl.edu (S.D.); ali.zarrinpar@surgery.ufl.edu (A.Z.)

**Keywords:** hepatocellular carcinoma, sorafenib resistance, CXCR6, CXCL16, CXCR6 antagonist, SBI457, β-catenin, targeted therapy, tumor microenvironment, precision oncology

## Abstract

**Background/Objectives**: Hepatocellular carcinoma (HCC) therapies are limited by poor response, rapid resistance, and recurrence of aggressive disease. Sorafenib, a multi-tyrosine kinase inhibitor, can trigger β-catenin stabilization and activation, contributing to resistance. Overexpression of the chemokine receptor CXCR6 and its ligand CXCL16 and hyperactivation are implicated in HCC progression and β-catenin stabilization. We hypothesized that SBI-457, a small-molecule CXCR6 antagonist we developed, could disrupt CXCR6/β-catenin crosstalk and enhance sorafenib sensitivity. **Methods**: We tested SBI-457 alone and in combination with sorafenib in SK-Hep-1 xenograft models and a panel of human HCC cell lines. Tumor burden, β-catenin activation, and CXCR6 expression were assessed by tumor volume measurements, immunohistochemistry, Western blotting, and immunofluorescence. Soluble CXCL16 levels were quantified by ELISA, and cell death responses were evaluated using MTT assays. **Results**: In vivo, SBI-457 combined with sorafenib reduced normalized tumor volume by 55% compared to vehicle controls, modestly exceeding monotherapy effects, and attenuated sorafenib-induced β-catenin upregulation. In vitro, SBI-457 blocked nuclear accumulation of β-catenin and reversed sorafenib-induced increases in β-catenin levels. Enhanced cell death was observed in specific “responder” HCC cell lines (Hep-3B, SNU-398, JHH-5), which correlated with high intracellular β-catenin, secretion of soluble CXCL16, and expression of a high molecular weight form of CXCR6. In contrast, “non-responder” cell lines with conventional CXCR6 expression and low CXCL16 secretion showed no enhanced cell death response. **Conclusions**: CXCR6 antagonism with SBI-457 can modulate β-catenin activation and may help overcome sorafenib resistance in selected HCC models. These findings support further development of CXCR6 antagonists as single agents or combination therapies to improve treatment outcomes in HCC.

## Simple Summary

Liver cancer is one of the deadliest cancers worldwide, and many patients are treated with the drug sorafenib. Unfortunately, this treatment often stops working because the cancer finds ways to resist it. In this study, we focused on a receptor abundant on the surface of liver cancer cells called CXCR6, which communicates with signals in the tumor environment and helps cancer cells survive. We developed a new compound, SBI-457, that blocks CXCR6. Using models of liver cancer, we found that this compound reduced tumor growth and prevented the buildup of a protein called β-catenin, which is linked to drug resistance. Importantly, we also discovered that only certain types of liver cancer cells responded strongly, depending on their specific features related to CXCR6 and β-catenin. These findings suggest that blocking CXCR6 may make sorafenib more effective and could guide personalized treatment approaches for patients with liver cancer.

## 1. Introduction

Current leading therapies such as sorafenib and immunotherapies for liver cancer are limited by low response, resistance, and recurrence of a more aggressive disease [1]. The chemokine receptor CXCR6 and its ligand CXCL16 have emerged as key players in cancer biology, influencing both immune cell recruitment and intrinsic cellular processes [2]. While their role in shaping the immune microenvironment is under active investigation, their contribution to proliferation, metastasis, and promoting therapeutic resistance remains poorly understood. Clinical studies in hepatocellular carcinoma (HCC) have previously linked elevated CXCR6 expression with aggressive disease and poor survival [3,4,5]. Blocking CXCR6 expression with shRNA has demonstrated reduced tumor growth, cytokine secretion, invasiveness, and metastasis in xenograft studies, validating CXCR6, as a therapeutic target in HCC [3].

We previously developed SBI-457, a selective, orally bioavailable CXCR6 antagonist with favorable pharmacokinetics and antitumor activity in xenograft models [6]. To advance its development, we wanted to understand the downstream effects of CXCR6 antagonism and how it may complement current HCC therapies. Sorafenib, a multi-tyrosine kinase inhibitor, has been a standard first-line therapy for advanced HCC but is limited by modest efficacy and rapid development of resistance [7,8]. Although newer immunotherapy-based regimens have expanded treatment options, resistance and immune-related toxicity remain major barriers [9,10,11,12]. Combination therapies offer a multipronged approach to target the tumor microenvironment and a potential for precision therapies based on validated biomarkers and modulation of key intracellular pathway that drive therapy resistance and aggressive forms of HCC [1].

**Scheme 1:**
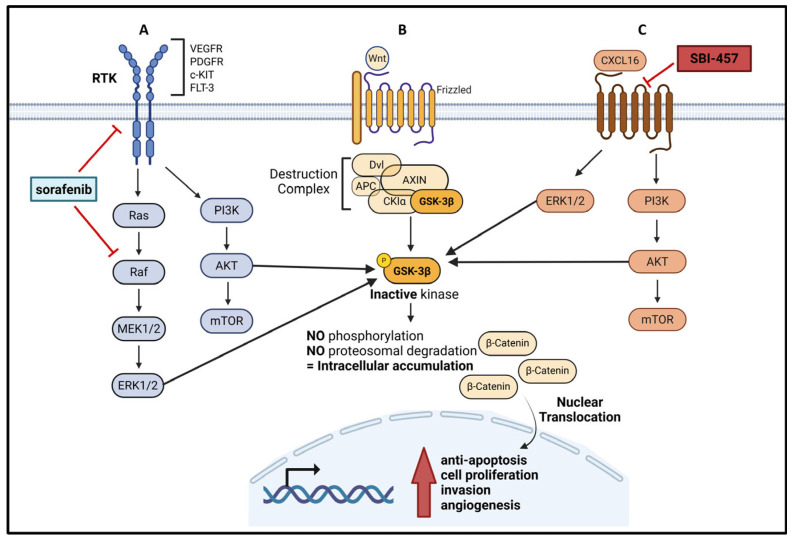
Molecular pathways contributing to sorafenib resistance. (A) Sorafenib resistance. (B) Aberrant β-catenin signaling. (C) Hyperactive CXCR6.

Sorafenib resistance in HCC arises through multiple mechanisms, including alterations in apoptosis signaling, epithelial–mesenchymal transition, oxidative stress adaptation, autophagy, and activation of compensatory pathways such as PI3K/AKT, JAK/STAT, and MAPK cascades [13,14,15,16,17,18,19,20,21]. These mechanisms have been extensively reviewed elsewhere; therefore, in this study we focus specifically on the role of CXCR6 in regulating β-catenin activation as a potential contributor to sorafenib resistance [22,23,24]. The Wnt/β-catenin pathway, dysregulated in up to 50% of HCCs, is strongly associated with immune evasion and sorafenib resistance [25]. In addition, sorafenib treatment can also lead to activation and nuclear accumulation of β-catenin through dysregulation of downstream kinases [26,27]. Despite the development of several preclinical small-molecule inhibitors, there is no clinical agent to date to directly target β-catenin and overcome resistance and improve response to therapy [28,29]. β-catenin is considered undruggable, possibly due to its shallow ill-defined binding pockets, overlapping binding interfaces with many critical proteins [30,31], and toxicity due to its critical role in tissue homeostasis [32,33]. Indirect modulation through upstream effectors, such as CXCR6, may provide a safer and more effective strategy. Prior studies suggest crosstalk between CXCR6 signaling and β-catenin activation [34], potentially through GSK3β inactivation promoting stabilization of β-catenin by preventing its phosphorylation and proteasomal degradation (Scheme 1). Following escape from degradation, β-catenin can translocate to the nucleus and activate transcription of proteins that promote drug resistance, anti-apoptosis, cell proliferation, angiogenesis, and metastasis [35].

We hypothesized that SBI-457, our CXCR6 antagonist, could block this crosstalk, thereby attenuate β-catenin (CTNNB1)-mediated resistance and enhance the activity of sorafenib. To test this, we evaluated the effects of SBI-457 alone and in combination with sorafenib in xenograft models and across a panel of HCC cell lines. We further investigated whether β-catenin activation, soluble CXCL16 secretion, and CXCR6 expression predicted treatment response. Our results indicate that CXCR6 antagonism with SBI-457 can modulate β-catenin activation and may improve response to sorafenib in select HCC cells that have high expression of β-catenin, shed soluble CXCL16, and have a high-molecular-weight isoform of CXCR6. These results have important implications for further development of CXCR6 antagonists and their use in combination treatments of HCC.

## 2. Methods

### 2.1. Cell Culture

Human HCC cell lines (SK-Hep-1, Hep-3B, SNU-398, JHH-2, and JHH-5) were obtained from ATCC or JCBR Cell Bank based on reported CXCR6 or b-catenin expression. Cells were cultured in Dulbecco’s Modified Eagle Medium (DMEM), Minimum Essential Medium (MEM), RPMI-1640, or Williams E Medium w/GlutaMAX™ (as appropriate for each line) supplemented with 10% fetal bovine serum (FBS) and 1% penicillin–streptomycin at 37 °C in a humidified atmosphere containing 5% CO_2_. ValiScreen^®^ CXCR6 expressing CHO-K1 (Revvity, Waltham, MA, USA; #ES-720-C) was maintained in F12 Ham’s medium supplemented with 800 μg/mL G418 (ThermoFisher, Waltham, MA, USA; #10131035). Experiments were performed at passages 6 to 10.

### 2.2. Xenograft Model

All animal experiments were conducted in accordance with the National Institutes of Health Guide for the Care and Use of Laboratory Animals approved by the University of Florida Institutional Animal Care and Use Committee (IACUC Protocol #202300000559). NOD.Cg-Prkdc^scid^ Il2rg^tm1Wjl^/SzJ (RRID:IMSR_JAX:005557) were purchased from JACS Laboratory and maintained as a breeding colony at UF. Six- to eight-week-old male NSG (NOD.Cg-Prkdc^scid^ Il2rg^tm1Wjl^/SzJ) mice were used for subcutaneous xenograft models. The number of mice was determined based on a sample size calculation assuming a large effect size (0.8), which yielded an estimated statistical power of 0.63 for the selected group size. SK-Hep-1 cells (1 × 10^7^) suspended in 50 µL MEM and 50 µL basement membrane extract (BME) were injected subcutaneously into the right flank. Mice were randomly assigned to experimental groups without any selection bias for specific treatments, and the order of treatment and assessment was not predetermined or influenced by group assignment. Mice (*n* = 3–5 per group) received vehicle, sorafenib (30 mg/kg), SBI-457 (30 mg/kg), or combination therapy (30 mg/kg each) every day or every other day in the case of sorafenib (both monotherapy and dose in combination). Treatment started four days post-injection via oral gavage and continued for 42 days. Tumor volumes were measured every other day with digital calipers and calculated as (length × width^2^)/2. Mice were weighed at each measurement to monitor toxicity. At the endpoint, tumors were excised, weighed, and fixed for histology or snap-frozen for protein analysis. Mice were maintained in a specific-pathogen-free (SPF) facility at the University of Florida. Animals were housed in individually ventilated cages (maximum of five per cage) with corncob bedding and nesting material for environmental enrichment. The animal room was maintained on a 12 h light/dark cycle at 22 ± 2 °C with 40–60% relative humidity. Standard laboratory chow and water were provided ad libitum. Animals were monitored daily by trained veterinary staff for general health, behavior, and signs of distress. Body weight and activity were assessed throughout this study. Humane endpoints were predefined, and animals exhibiting these signs were humanely euthanized according to IACUC-approved guidelines.

### 2.3. Drug Treatment and Viability Assays

Sorafenib (TOCRIS) and SBI-457 [6] were dissolved in DMSO for 10 mM stock solutions and diluted in culture medium to the desired concentration (final DMSO < 0.5%). For cell viability assays, cells were seeded in 96-well plates (0.01 × 10^6^ cells/well). Following overnight adherence, cells were pretreated with SBI-457 for 15 min and then sorafenib (Tocris, Bristol, United Kingdom #284461-73-0) for 24–72 h, after which cell viability was assessed by MTT assay (PROMEGA, Madison, WI, USA #G4100) according to the manufacturer’s instructions. Absorbance was measured at 570 nm using a microplate reader. GI50 and Emax values were derived by nonlinear regression using GraphPad Prism 10.2.2, Boston, MA, USA.

### 2.4. Protein Extraction and Western Blotting

Tumors and cell pellets were lysed in RIPA buffer containing protease and phosphatase inhibitors. For nuclear and cytoplasmic fractionation, cells were processed with NE-PER Nuclear and Cytoplasmic Extraction Reagents (ThermoFisher, #78833). Protein concentrations were determined using a Pierce ™ BCA kit (ThermoScientific #23227). Equal amounts (20–40 µg) of protein were separated by SDS-PAGE and transferred to PVDF membranes. Membranes were blocked in 5% nonfat milk and incubated with primary antibodies against CXCR6 (Invitrogen/ThermoFisher, Waltham, MA, USA; #PA5-19936, 1:500), anti-β-catenin (Cell Signaling Technology, Danvers, MA, USA #9582; 1:500), *p*CREB (Cell Signaling Technology, #9198S; 1:500), GSK3β (Cell Signaling Technology, #9832; 1:1000), p-GSK3β (Cell Signaling Technology, #9336; 1:1000), and GAPDH (Invitrogen #39-8600; 1:1000) or Histone H2A.X (Thermofisher, Waltham, MA, USA #10856-1-AP; 1:1000) as loading controls. After incubation with HRP or Alexa Fluor™ conjugated secondary antibodies, signals were visualized by chemiluminescence or fluorescence, respectively. Band intensities were detected and quantified using ChemiDoc MP Imaging System (BIO-RAD, Hercules, CA, USA) and Image Lab software or Odyssey^®^ CLx Imager (LI-COR Biosciences, Lincoln, NE, USA). The uncropped blots are shown in Figures S1, S2 and S4 of File S1.

### 2.5. Immunohistochemistry and Immunofluorescence

Formalin-fixed paraffin-embedded (FFPE) tumor sections (5 µm) were stained with hematoxylin and eosin (H&E) or subjected to immunohistochemistry (IHC) for Ki67. For IHC, antigen retrieval was performed in citrate buffer, endogenous peroxidase activity was quenched with hydrogen peroxide, and sections were incubated with anti-Ki67 antibody (Cell Signaling, #9027; 1:250). Detection was performed with ImmPRESS horse anti-rabbit IgG, followed by chromogen development using 3,3′-Diaminobenzidine (DAB) (Vector Laboratories, Newark, CA, USA) and counterstained with hematoxylin. The number and percentage of Ki67-positive cells were quantified using QuPath from at least five random high-power fields per tumor.

For immunofluorescence, SK-Hep-1 cells were seeded at 0.02 × 10^6^ per well in collagen-coated Greiner µClear imaging plates (#7000190) and stimulated with specified treatments after 24 h adherence. Cells were fixed in paraformaldehyde, permeabilized, and incubated with anti-β-catenin antibody followed by fluorophore-conjugated secondary antibody Alexa Fluor 488 (ThermoScientific, Waltham, MA, USA; #A32731TR). Nuclei were counterstained with DAPI. Slides were imaged by fluorescence microscopy, and nuclear versus cytoplasmic localization was evaluated.

### 2.6. ELISA for Soluble CXCL16

Cells were seeded 0.3 × 10^6^ in 12-well plates and treated as indicated. Culture supernatants were collected at 2 h and 24 h and clarified by centrifugation. Soluble CXCL16 concentrations were quantified by enzyme-linked immunosorbent assay (Invitrogen, Cat. No. EHCXCL16) following the manufacturer’s instructions. Values were normalized to cell number.

### 2.7. Statistical Analysis

Data are presented as mean ± SD. One-way ANOVA with Tukey’s post hoc test (parametric) or Kruskal–Wallis with Dunn’s test (non-parametric) was used to compare groups. Spearman correlations assessed associations between protein expression and tumor parameters. Analyses were performed using GraphPad Prism 10.2.2, with significance set at * *p* < 0.05. For ANOVA tests with Bonferroni or Tuckey’s correction, one star (* *p* < 0.05) through four stars (**** *p* < 0.0001) denote increasing statistical significance in ANOVA-derived pairwise comparisons whose *p*-values have been Bonferroni-adjusted or Tukey’s post-hoc test applied to control the family-wise error rate.

## 3. Results

### 3.1. CXCR6 Antagonism with SBI-457 Reduces Tumor Growth In Vivo

**Figure 1:**
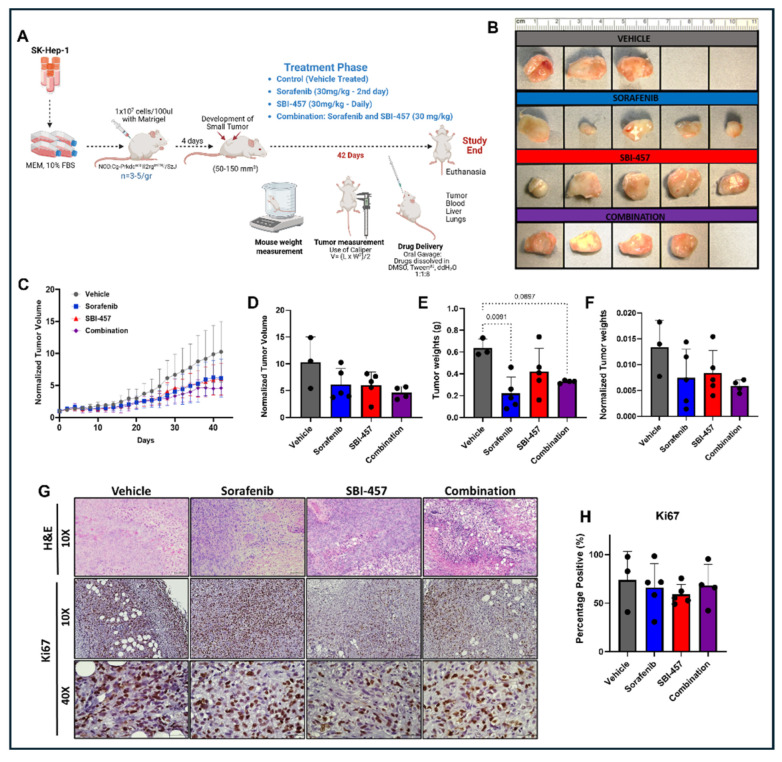
**Combination treatment of sorafenib and SBI-457 decreases tumor burden.** (**A**) Schematic of experimental workflow for in vivo analysis of efficacy of sorafenib and SBI-457 singularly and in combination. (**B**) Tumor xenograft models were constructed with SK-HEP-1 cells, and mice were euthanized at the completion of six weeks of treatment, after which subcutaneous tumors were isolated and photographed (*n* = 3–5). (**C**) The subcutaneous tumor growth was measured and recorded every other day, and tumor volume was calculated using (mm^3^)  =  (L × W^2^)/2, where L is the long axis and W the short axis. Growth curve showing normalized tumor volumes across treatment groups over the course of this study (42 days). (**D**) Endpoint normalized tumor volumes. (**E**) Endpoint tumor weights (comparison performed by one-way ANOVA). (**F**) Normalized tumor weights (endpoint tumor weights were normalized to starting tumor volumes). (**G**) Immunohistological assessment of tumors via H&E and immunohistochemistry for Ki67. A scale bar of 100 µm and 50 µm were used for 10× and 40× magnifications, respectively. (**H**) Comparison of percentage of cells in tumors at endpoint that were positive for Ki67. Values are reported as mean + SD (*n* = 3–5). All data in this figure was determined to be parametric, and an ordinary one-way ANOVA test followed by the Tukey’s posttest for multiple comparisons was run with statistical significance assigned as *p* < 0.05.

SK-Hep-1 xenografts were treated with vehicle (*n* = 3), sorafenib (*n* = 5), SBI-457 (*n* = 5), or combination (*n* = 4) therapy for 42 days (see Section 2.2 in Section 2 and Figure 1A). All groups maintained stable weights without evidence of toxicity (File S1). At the endpoint, tumors from sorafenib- and SBI-457-treated mice were visibly smaller than controls, with the combination group showing the greatest reduction in tumor size (Figure 1B). Because starting tumor volumes varied modestly across treatment groups, we included an additional analysis where endpoint tumor weights were divided by the initial tumor volume at randomization. This calculation was performed solely to illustrate that the relative treatment effects on tumor burden remained consistent after accounting for baseline tumor size differences. Normalized tumor volumes were reduced by 40.0% with sorafenib, 41.5% with SBI-457, and 55.1% with the combination relative to vehicle (Figure 1C,D). Tumor weights were significantly reduced with sorafenib (*p* = 0.0091) and trended lower with the combination (*p* = 0.0697) (Figure 1E). After normalization of growth rate and treatment response between and within experimental groups, the combination was more effective than either monotherapy, though not statistically significant (Figure 1F; File S1). Histology revealed no differences in morphology (Figure 1G). Ki67 staining showed no significant changes, though SBI-457 monotherapy had the lowest proportion of proliferating cells (Figure 1H; File S1).

### 3.2. SBI-457 Attenuates Sorafenib-Induced β-Catenin Activation and Modulates GSK3β

**Figure 2:**
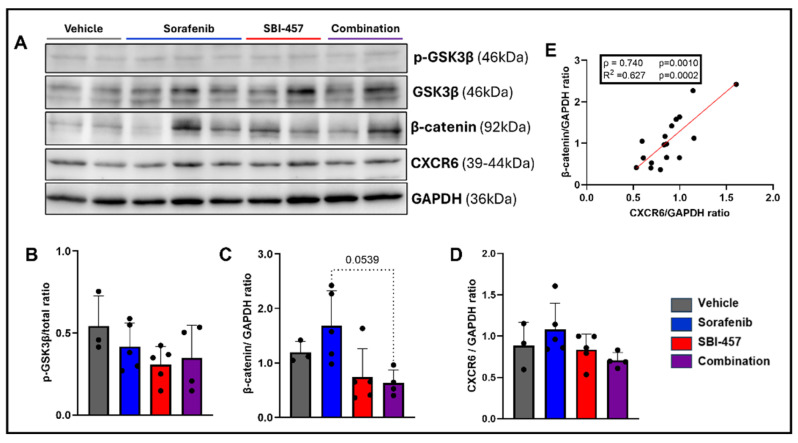
**Combination therapy attenuated the sorafenib-induced increase in β-catenin expression.** (**A**) Representative western blot of tumors treated with vehicle (gray), sorafenib (blue), SBI-457 (red), or combination (purple) for proteins of interest (p-GSK3β, GSK3β, β-catenin, and CXCR6) and housekeeping GAPDH. All samples were analyzed over two Western blot membranes per technical replicate. Membranes were probed and imaged simultaneously. Western blot analysis and quantification of the (**B**) ratio of phosphorylated GSKβ to total GSKβ, (**C**) β-catenin, and (**D**) CXCR6. (**E**) Spearman correlation between β-catenin and CXCR6 protein levels in tumors at endpoint. Red line represents a best fit of a linear regression. Values are reported as mean + SD (*n* = 3–5). All data in this figure was determined to be non-parametric, and a Kruskal–Wallis ANOVA followed by the Dunn’s multiple comparisons test was run with statistical significance assigned as *p* < 0.05.

Tumor lysates from all the treatment groups in the xenograft study were analyzed for CXCR6, β-catenin, and GSK3β protein levels using Western blotting (Figure 2A).Analysis of GSK3β, a central negative regulator of Wnt/β-catenin signaling, revealed a modest reduction in the ratio of phosphorylated GSK3β (inactive form) to total GSK3β in SBI-457-treated tumors, suggesting a trend toward increased GSK3β activity (Figure 2B; File S1). Sorafenib treatment increased β-catenin protein levels, whereas SBI-457 reduced them to baseline (Figure 2C, File S1). In the combination, SBI-457 attenuated sorafenib-induced β-catenin upregulation, trending toward significance (*p* = 0.054; Figure 2C). CXCR6 protein levels were also elevated in sorafenib-treated tumors (Figure 2D). Correlation analyses revealed that β-catenin levels correlated with tumor weight (ρ = 0.40) and volume (ρ = 0.60; File S1). Across all tumors, CXCR6 and β-catenin expression were strongly correlated (ρ = 0.74, *p* = 0.001; Figure 2E) and even more strongly in sorafenib-treated tumors (ρ = 0.90; File S1). Although differences were subtle and did not reach statistical significance, this trend supports a mechanism by which CXCR6 antagonism prevents sorafenib-induced β-catenin stabilization through maintenance of GSK3β activity.

### 3.3. SBI-457 Blocks Nuclear Accumulation of β-Catenin in SK-Hep-1 Cells

**Figure 3:**
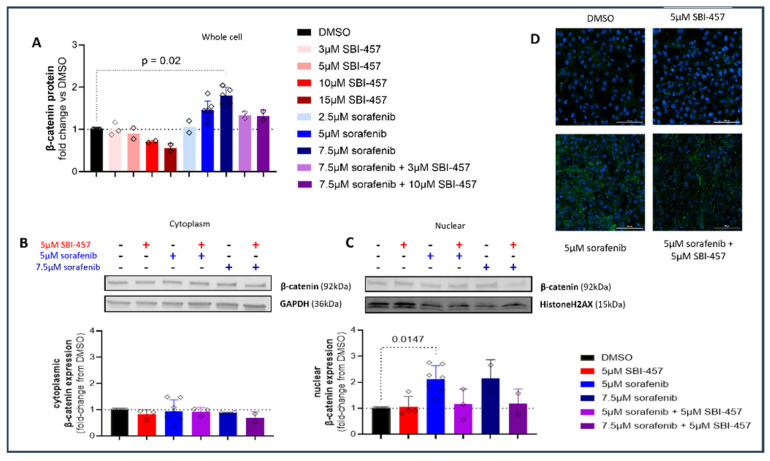
**SBI-457 attenuates sorafenib-induced nuclear accumulation of β-catenin in SK-Hep-1 cells.** Whole-cell (**A**) and nuclear and cytoplasmic (**B**,**C**) Western Blot analyses and quantification of sorafenib (blue)-, SBI-457 (red)-, or combination (purple)-treated SK-Hep-1 cell lysates at 48 h. Data shown are averages + SD. (**D**) Confocal immunofluorescence microscopy of SK-Hep-1 cells treated with sorafenib (5 µM), SBI-457 (5 µM), or combination at 24 h. β-catenin was stained in green color, and cell nuclei were counterstained with DAPI (blue). Scale bar: 100 µm.

In vitro Western blot analysis showed that sorafenib induced a dose-dependent increase in total β-catenin, while SBI-457 alone decreased β-catenin compared to untreated control (Figure 3A). Further analysis showed that cytoplasmic β-catenin levels remained unchanged across all treatments (Figure 3B). However, nuclear fractionation showed that sorafenib significantly increased nuclear β-catenin levels (>2-fold at 5 μM; *p* = 0.0125), while cotreatment with SBI-457 inhibited this increase and kept nuclear β-catenin levels at baseline values (Figure 3C, Figures S3 and S5 supplemental data.docx). Immunofluorescence imaging qualitatively confirmed a sorafenib-induced increase in total β-catenin accumulation and its inhibition by SBI-457 in SK-Hep-1 cells (Figure 3D).

### 3.4. CXCR6 Antagonism Enhances Sorafenib-Mediated Cell Death in Select HCC Cell Lines

**Figure 4:**
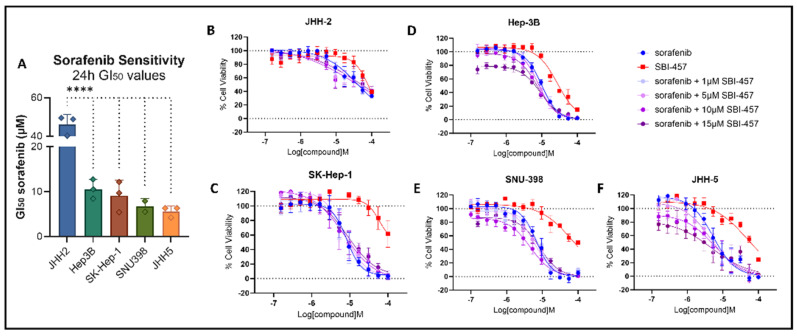
**Correlation of treatment-related cell death in HCC cells to CXCR6 expression and signaling.** (**A**) Growth inhibitory (GI50) values following 24 h sorafenib treatment across five-HCC-cell-line panel (ANOVA w/Bonferroni adjustment, **** *p* < 0.0001; each point represents a biological replicate). (**B**–**F**) Sorafenib, SBI-457, and combined effect in sorafenib cell death (MTT) dose–response shift following 24 h compound treatments. Data represented as + SD; each point represents an average of three biological replicates.

MTT-based cell viability assays across five HCC cell lines revealed differential responses. Sorafenib alone induced dose-dependent cytotoxicity in all cell lines except JHH2 where complete cell death was not seen at the highest concentration of sorafenib (Figure 4A). SBI-457 alone had minimal effects on cell viability (Figure 4B–F). In SK-Hep-1 and JHH2 cells, SBI-457 did not enhance sorafenib-induced cell death (Figure 4B,C). However, in Hep-3B, SNU-398, and JHH-5 cells, SBI-457 decreased maximal viability at sub-GI50 sorafenib concentrations, indicating enhancement of cell death or response to sorafenib (Figure 4D–F). Thus, these were classified as “responder” cell lines, while SK-Hep-1 and JHH-2 were termed “non-responders”. We also noted that JHH5, which was most sensitive to sorafenib-mediated cell death among the cell lines tested, showed the best response to combination treatment with SBI-457.

### 3.5. Response to Combination Therapy Correlates with CXCL16 Secretion and CXCR6 Isoform Expression

**Figure 5:**
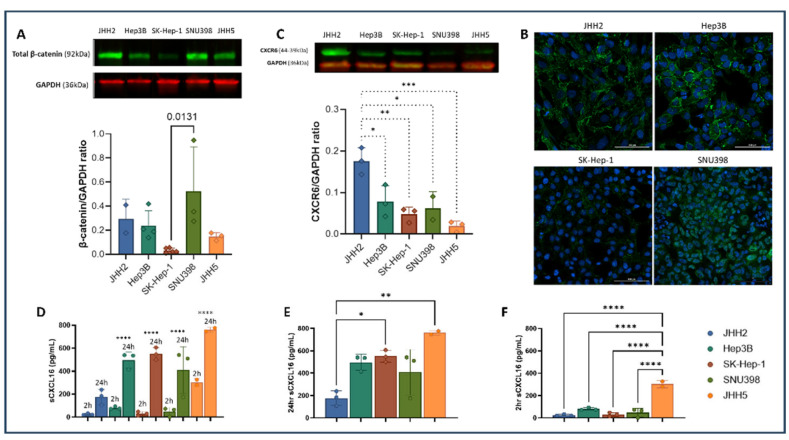
**Correlation of treatment-related cell death in HCC cells to CXCR6, soluble CXCL16, and β-catenin expression.** Total β-catenin protein expression across HCC cells assessed in (**A**) Western blot and (**B**) immunofluorescence imaging at 24 h. (**C**) CXCR6 protein expression assessed via Western blot. One-way ANOVA, Tukey’s multiple comparisons test; each point represents a biological replicate. (**D**–**F**) Soluble CXCL16 secretion at 2 h (**E**) and 24 h (**F**) across five-HCC-cell-line panel as detected in ELISA. Two-way ANOVA, Tukey’s multiple comparisons test; each point represents a biological replicate run in duplicate (* *p* < 0.05, ** *p* < 0.01, *** *p* < 0.001, **** *p* < 0.0001). All data represented as mean + SD.

Baseline protein analysis revealed that JHH-2, Hep-3B, SNU-398, and JHH-5 expressed higher total β-catenin than SK-Hep-1 (Figure 5A). However, total β-catenin was not prognostic of the observed treatment-related cell death response. Western blotting identified CXCR6 at ~40 kDa in all lines, with JHH-2 showing the highest expression (Figure 5C). Intriguingly, the responder cell lines exhibited an additional ~75 kDa high-molecular-weight (MW) CXCR6 band (File S1), with JHH-5 showing the strongest expression. ELISA assay of the extracellular media demonstrated that all cell lines shed increasing levels of soluble CXCL16 (sCXCL16), with JHH-5 secreting significantly higher levels of CXCL16 than others at both 2 h and 24 h (**** *p* < 0.0001; Figure 5D–F, File S1). Notably, high extracellular sCXCL16 levels correlated with high-MW CXCR6 expression and enhanced cell death in responder cells due to combination treatment. Collectively, these data suggest that high sCXCL16 secretion and high expression of high-MW CXCR6 isoforms may underlie differential responses to CXCR6 antagonism and sorafenib-mediated cell death.

## 4. Discussion

As a step forward in the development of SBI-457-based CXCR6 antagonists, this study explores CXCR6 antagonism as a potential strategy to block crosstalk with sorafenib and β-catenin pathways and to increase response and overcome resistance to sorafenib in hepatocellular carcinoma [6]. In the SK-Hep-1-derived xenograft model, the CXCR6 antagonist, SBI-457, modestly reduced tumor burden alone and non-significantly in combination with sorafenib while attenuating sorafenib-induced β-catenin upregulation. In SK-Hep-1 cells, SBI-457 blocked the nuclear accumulation of β-catenin, the transcriptionally active pool. Screening across a panel of HCC cell lines revealed differential responses, with enhanced sorafenib-mediated cell death in Hep-3B, SNU-398, and JHH-5 (“responders”) but not in SK-Hep-1 or JHH-2 (“non-responders”) [36]. Responsiveness correlated with secretion of soluble CXCL16 and expression of a higher-molecular-weight form of CXCR6, implicating extracellular remodeling and receptor dysregulation in modulating drug sensitivity.

### 4.1. CXCR6/β-Catenin Crosstalk and Resistance

The Wnt/β-catenin pathway is dysregulated in up to 50% of HCCs and strongly associated with resistance to both sorafenib and immunotherapies [37,38,39,40]. Sorafenib itself has been reported to trigger adaptive β-catenin activation through stabilization and nuclear translocation [26]. Our findings confirm this effect, showing that sorafenib increased β-catenin expression in xenografts (Figure 2C) and SK-Hep-1 cells (Figure 3). Importantly, SBI-457 reversed this increase, supporting mechanistic crosstalk between CXCR6 and β-catenin signaling. The observed strong correlation between CXCR6 and β-catenin protein levels across tumors further strengthens this link (Figure 2E).

### 4.2. CXCR6/GSK3β/β-Catenin Crosstalk

Our results further suggest that the CXCR6 pathway contributes to sorafenib resistance by modulating GSK3β activity. Phosphorylation of GSK3β at Ser-9 is known to inactivate the kinase, leading to β-catenin stabilization and nuclear accumulation [41,42,43]. Prior studies have linked CXCR6/CXCL16 hyperactivation to increased GSK3β phosphorylation [34]. In our xenograft model, SBI-457 treatment modestly reduced the phosphorylated-to-total GSK3β ratio, consistent with preservation of GSK3β activity and subsequent downregulation of β-catenin (Figure 2B). While these effects were not statistically significant, they were directionally consistent across replicates and aligned with the observed attenuation of β-catenin nuclear translocation. Together, these findings support our initial hypothesis (Schematic 1) in which CXCR6 antagonism prevents sorafenib-induced β-catenin stabilization by maintaining GSK3β activity. By blocking nuclear accumulation of β-catenin, CXCR6 antagonism may provide an indirect way to modulate activity of the otherwise “undruggable” β-catenin pathway.

### 4.3. Differential Responses Across HCC Models

Although SK-Hep-1 xenografts provided evidence that SBI-457 attenuates β-catenin activation, tumor regression was modest and not statistically significant (Figure 1B–F). This likely reflects the limited β-catenin dependency of SK-Hep-1 cells, which others have classified as a non-responder to β-catenin inhibition [36,44,45,46]. Additionally, viral factors such as HBx protein have been implicated for this lack of response to targeted therapies in hepatoma cells [45,46,47]. In contrast, Hep-3B, SNU-398, and JHH-5 cells, deemed responders to b-catenin inhibition, showed enhanced sorafenib-mediated cell death when combined with SBI-457. These responder models shared two distinctive features: (1) secretion of higher levels of soluble CXCL16 (Figure 5D–F), and (2) expression of a high-molecular-weight (~75 kDa) form of CXCR6 (Supplemental Figure S3, see supplementaldata.docx). These findings suggest that CXCR6 dysregulation, driven by extracellular CXCL16 shedding and altered receptor isoforms, may sensitize tumors to CXCR6 antagonism.

Notably, while CXCR6 and β-catenin expression were strongly correlated in xenograft tumors, this relationship was not uniformly observed across all HCC cell lines. This likely reflects the intrinsic molecular heterogeneity of HCC, including variability in β-catenin mutation status, Wnt pathway activation, and CXCL16 signaling. Such diversity suggests that CXCR6–β-catenin coupling is context-dependent, underscoring the importance of identifying responsive subtypes where this pathway is dominant and may predict therapeutic response.

### 4.4. Experimental Rationale and Future in Vivo Validation

Our combination studies were performed in SK-Hep-1 xenografts, a model previously validated for CXCR6 expression and used to establish SBI-457 monotherapy efficacy. Building on this foundation, the current work was designed to assess translational feasibility of combining SBI-457 with sorafenib in the same system. While the combination attenuated β-catenin activation without producing significantly greater tumor regression, this finding highlighted an important limitation of the SK-Hep-1 model, i.e., its low β-catenin dependency. To address this, we extended our investigation in vitro across HCC lines with differing β-catenin activity and responsiveness, which revealed mechanistic heterogeneity and identified putative biomarkers of response. While SK-Hep-1 xenografts served as an appropriate model due to prior validation of CXCR6 expression and SBI-457 monotherapy efficacy, these cells were subsequently found to represent a “non-responder” phenotype with limited β-catenin dependency. As such, the absence of a statistically significant additive effect in this model does not exclude therapeutic potential in other HCC contexts. Ongoing studies are extending these experiments to responder models such as JHH-5 to validate the combination effects in β-catenin-driven tumors.

### 4.5. Implications for Biomarker-Guided Therapy

Our results suggest that soluble CXCL16 and CXCR6 isoform expression may serve as biomarkers for identifying patients most likely to benefit from CXCR6-targeted therapies [4,48]. In clinical practice, such biomarkers could be readily assayed through plasma CXCL16 levels or tumor immunoblotting, guiding patient selection for combination therapy. This aligns with a precision-oncology framework, where pathway activation signatures direct therapy choice [49,50]. Moreover, indirect modulation of β-catenin via CXCR6 may avoid toxicities associated with direct Wnt/b-catenin inhibition [29,51,52].

### 4.6. Limitations

This study has several limitations. First, the xenograft experiments were limited by small sample sizes and variability in starting tumor volumes, which reduced statistical power. Second, SK-Hep-1 xenografts may underestimate therapeutic benefit given their β-catenin independence and repressed transcriptional output despite nuclear accumulation. Third, although we identified a high-MW CXCR6 form correlated with drug response, its molecular identity and signaling role remain unknown. Fourth, although this work focused primarily on β-catenin-dependent signaling, CXCR6 has also been implicated in angiogenic regulation through modulation of VEGF expression [5]. Future studies will assess whether SBI-457 alters tumor vasculature or angiogenic marker expression, which could represent an additional mechanism contributing to therapeutic response. Finally, in vivo studies were performed in NSG mice, which lack T and NK cells where CXCR6 is normally expressed. Thus, the immune-modulatory contributions of CXCR6 antagonism could not be assessed.

### 4.7. Future Directions

Further work is needed to define the molecular basis of the high-MW CXCR6 isoform, including whether it represents receptor modification, dimerization, or altered post-translational regulation. Studies in patient-derived xenografts and β-catenin-dependent or responder models will clarify the therapeutic efficacy of CXCR6 antagonists. Incorporating immunocompetent models will also be critical to assess the therapeutic benefit of CXCR6 antagonism due to dual roles of CXCR6 in tumor-intrinsic signaling and immune recruitment. Ultimately, integrating SBI-457 into biomarker-guided combination regimens may provide a path to expand treatment responses in HCC.

## 5. Conclusions

This study demonstrates that antagonism of CXCR6 with SBI-457 may attenuate sorafenib-induced activation of β-catenin and enhance sorafenib-mediated cell death in selected HCC models. In vivo, SBI-457 reduced tumor burden and blocked sorafenib-driven nuclear accumulation of β-catenin, while in vitro screening revealed enhanced responses in HCC cell lines characterized by high soluble CXCL16 secretion and expression of a higher-molecular-weight CXCR6 isoform. These findings identify CXCR6 as both a therapeutic target and a biomarker axis that contributes in resistance to sorafenib.

The results support further investigation of CXCR6 antagonists as single agents or in biomarker-guided combination regimens to expand the efficacy of existing frontline therapies in HCC. Future studies in β-catenin-dependent and immune-competent models are warranted to validate these findings and define the translational potential of CXCR6-targeted therapies.

## 6. Patents

Peddibhotla S, Hershberger PM, Kirby RJ, Malany S, Smith LH, Maloney PR, Sessions H, Divlianska D, Pinkerton AB. Preparation of azabicyclononanes and diazabicyclononanes as CXCR6 inhibitors and methods of use. PCT Int. Appl. (2021), WO/2021/007208 20210114 Issued 14 January 2021; US20220402908 Issued 22 December 2022 [53].

## Data Availability

The data presented in this study are available from the corresponding author upon reasonable request.

## Supplementary Materials

The following supporting information can be downloaded at: https://www.mdpi.com/article/10.3390/cancers17233818/s1, File S1: Supplementary Materials.

## Abbreviations

ADAM	A Disintegrin and Metalloproteinase
ANOVA	Analysis of Variance
BCA	Bicinchoninic Acid
BME	Basement Membrane Extract
CDX	Cell-Line-Derived Xenograft
CHO	Chinese Hamster Ovary
CXCR6	C-X-C Chemokine Receptor 6
CXCL16	C-X-C Motif Chemokine Ligand 16
DAB	3,3′-Diaminobenzidine
DMSO	Dimethyl Sulfoxide
DMEM	Dulbecco’s Modified Eagle Medium
ELISA	Enzyme-Linked Immunosorbent Assay
FBS	Fetal Bovine Serum
FFPE	Formalin-Fixed Paraffin-Embedded
GI50	50% Growth Inhibition Concentration
GPCR	G Protein-Coupled Receptor
GSK3β	Glycogen Synthase Kinase 3 Beta
HCC	Hepatocellular Carcinoma
H&E	Hematoxylin and Eosin
HRP	Horseradish Peroxidase
IF	Immunofluorescence
IHC	Immunohistochemistry
LEF	Lymphoid Enhancer Factor
MEM	Minimum Essential Medium
MTT	3-(4,5-Dimethylthiazol-2-yl)-2-5-Diphenyltetrazolium Bromide
MW	Molecular Weight
NE-PER	Nuclear and Cytoplasmic Extraction Reagent
NK	Natural Killer
NSG	NOD scid gamma (mice)
PBS	Phosphate-Buffered Saline
pCREB	Phosphorylated cAMP Response Element-Binding Protein
PVDF	Polyvinylidene Difluoride
RIPA	Radioimmunoprecipitation Assay
RPMI	Roswell Park Memorial Institute Medium 1640
SBI-457	Small-Molecule CXCR6 Antagonist
sCXCL16	Soluble CXCL16
SDS-PAGE	Sodium Dodecyl Sulfate Polyacrylamide Gel Electrophoresis
SD	Standard Deviation
TCF	T-Cell Factor
tmCXCL16	Transmembrane CXCL16
