# Supplementary material for: Targeting CXCR6 Disrupts β-Catenin Signaling and Enhances Sorafenib Response in Hepatocellular Carcinoma
Source: Cancers (Basel). 2025 Nov 28;17(23):3818. doi: 10.3390/cancers17233818 (PMC12691426; doi:10.3390/cancers17233818)
Supplement: Supplementary file 1 [file cancers-17-03818-s001.zip › cancers-3919960-supplementary.pdf]

<u>File S1: Supplementary Materials</u>

<u>Figure S1:</u> Combination therapy attenuated the sorafenib-induced increase in β-catenin expression

WB: anti-p-GSK3beta (Set 1)

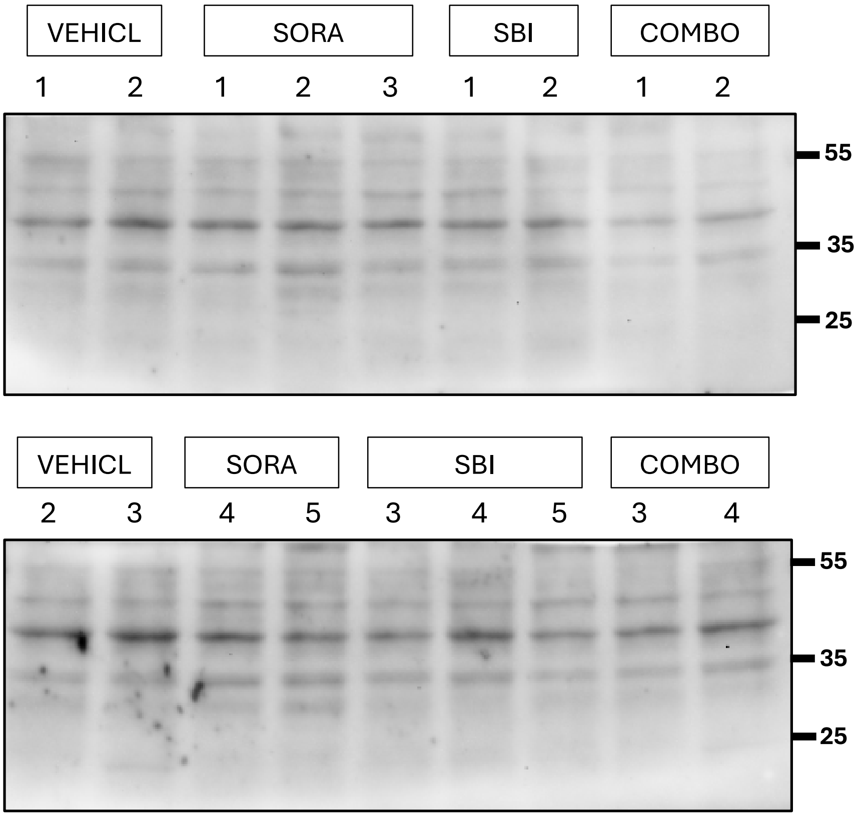

WB: anti-p-GSK3beta (Set 2)

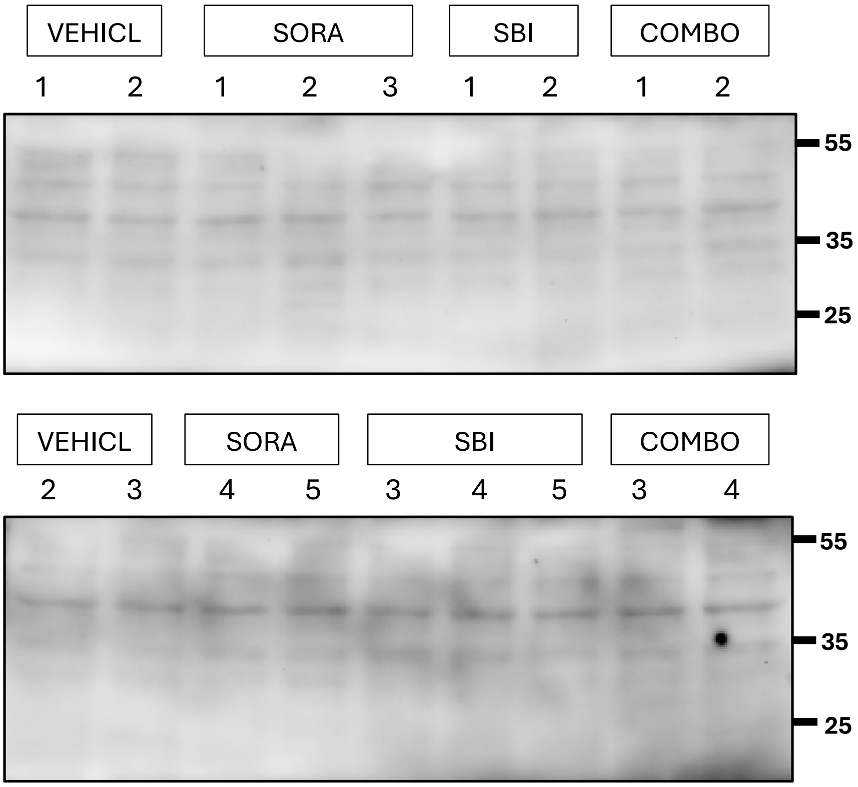

WB: anti-GSK3beta (Set 1)

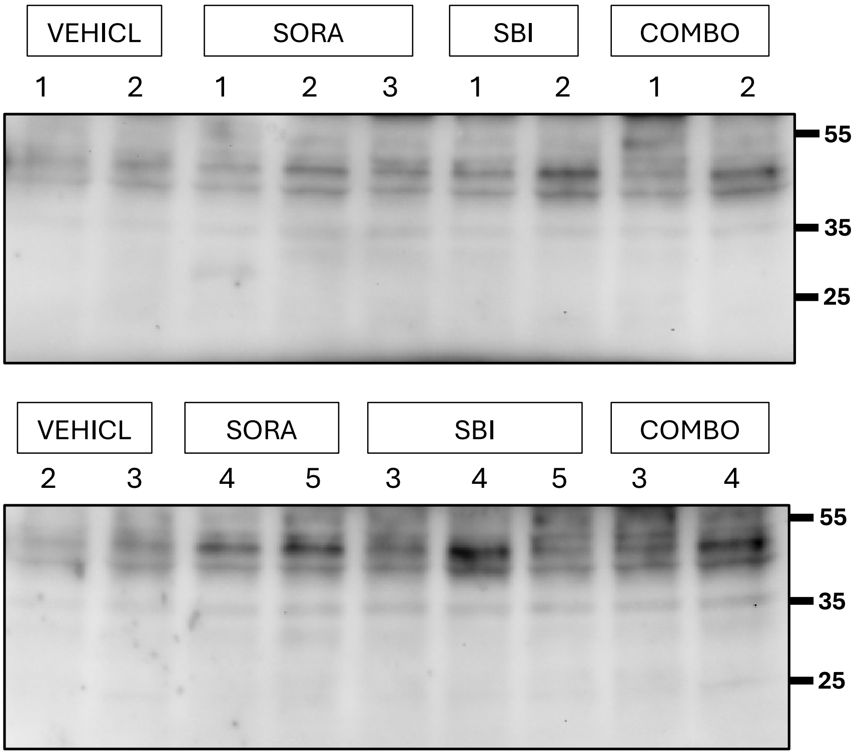

WB: anti-GSK3beta (Set 2)

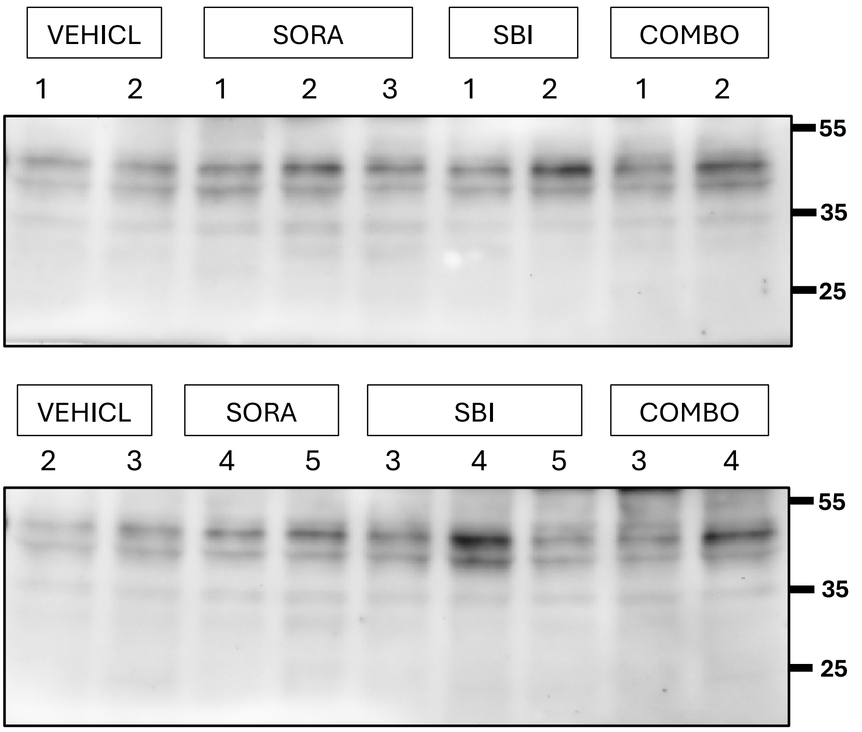

WB: anti-beta-catenin (Set 1)

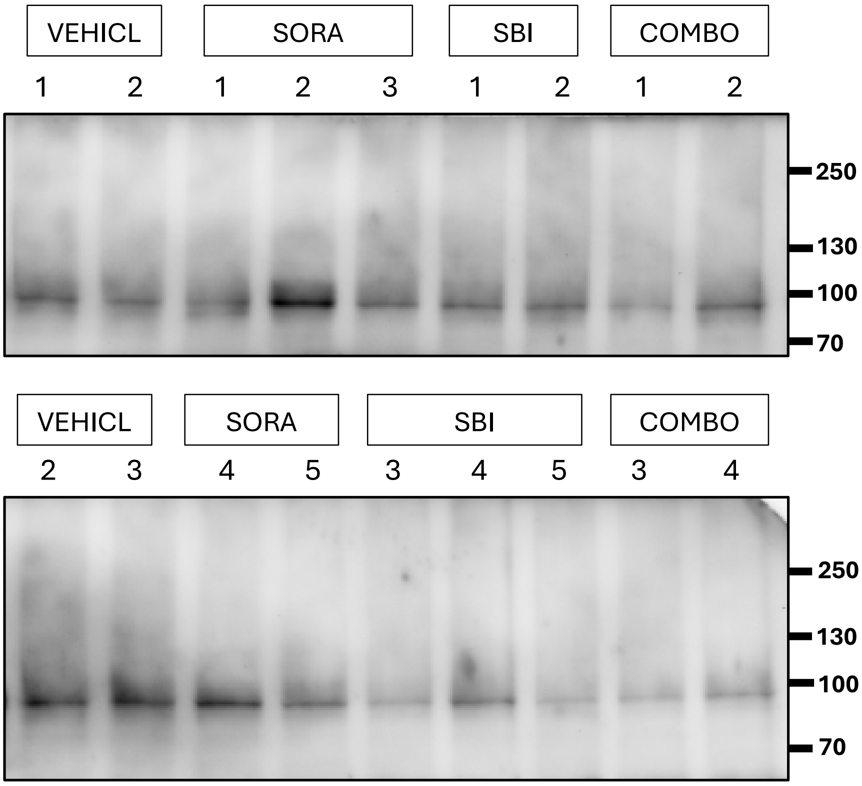

WB: anti-beta-catenin (Set 2)

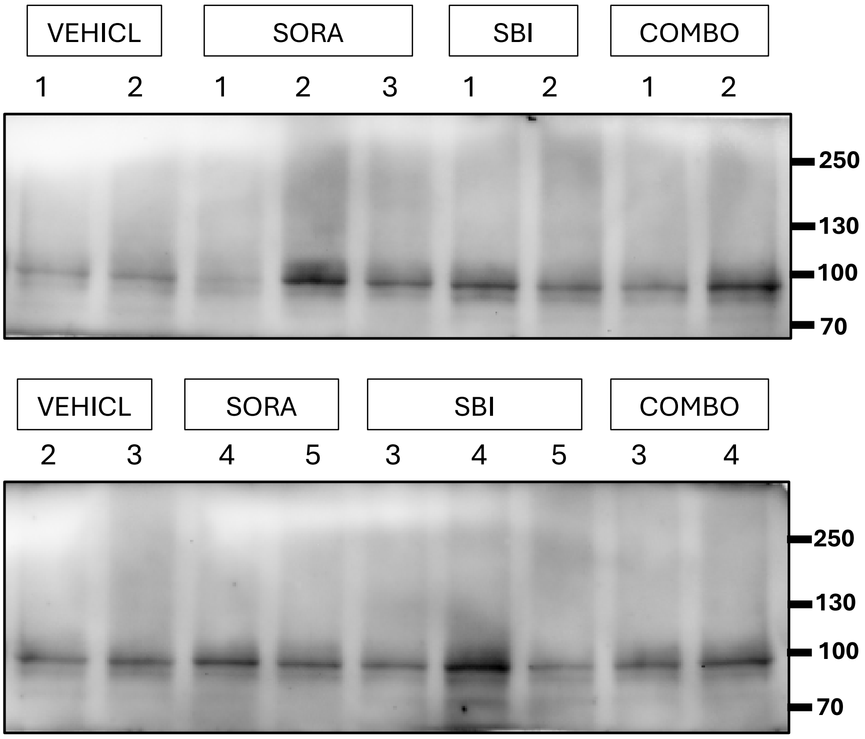

WB: anti-CXCR6 (Set 1)

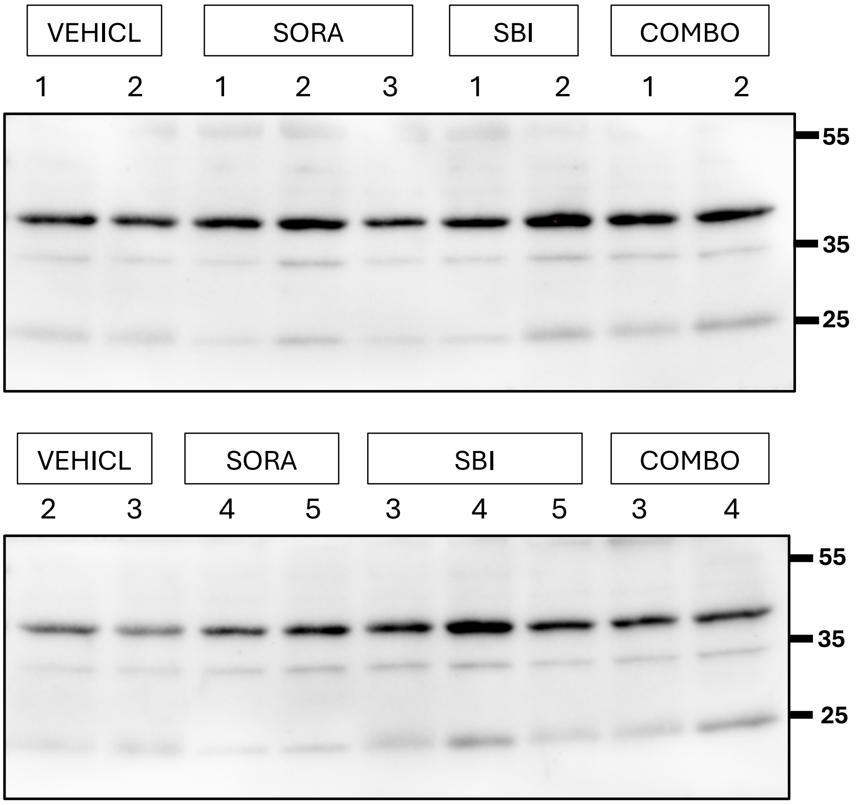

WB: anti-CXCR6 (Set 2)

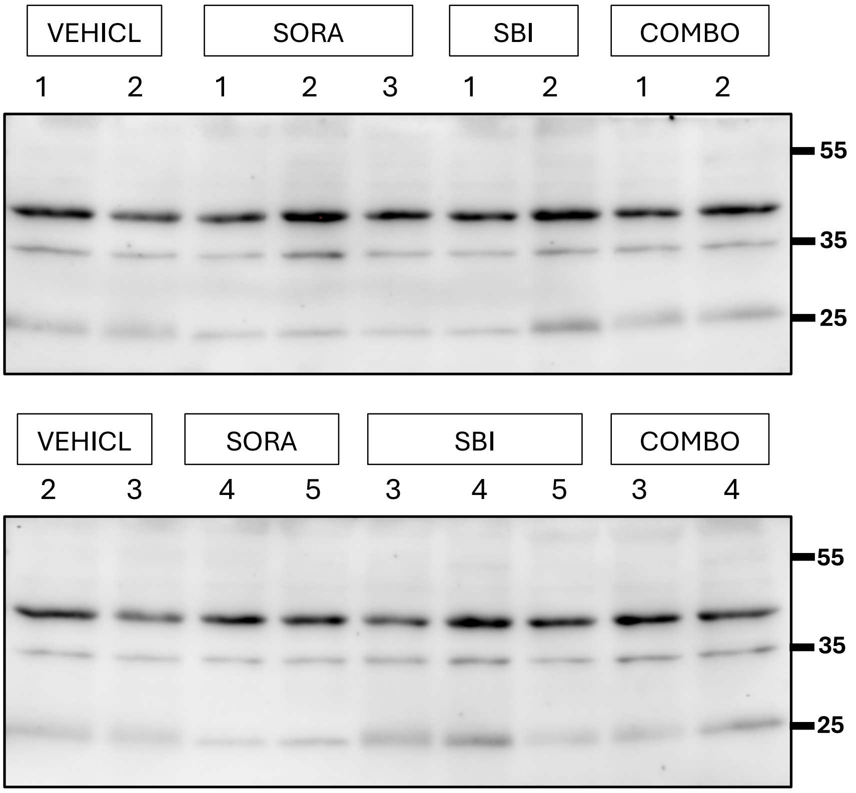

WB: anti-GAPDH (Set 1)

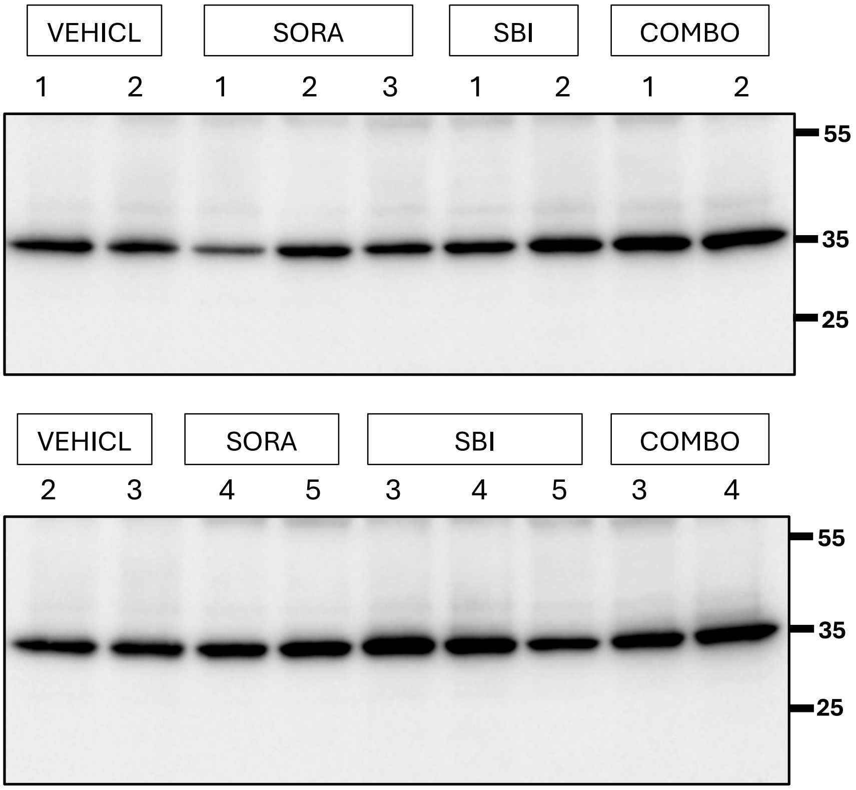

WB: anti-GAPDH (Set 2)

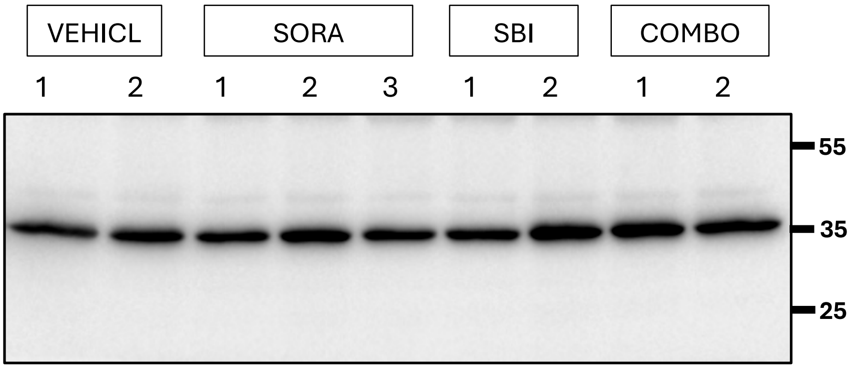

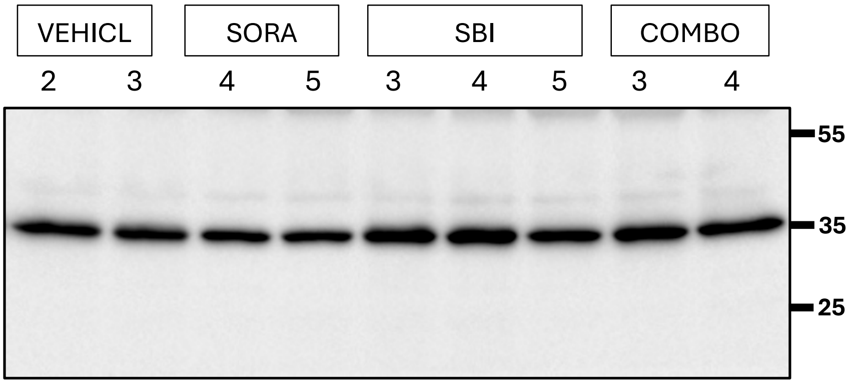

SET 1 (Raw Densitometry)																	
	CTRL			SORA					SBI					COMBO			
**protein**	**1**	**2**	**3**	**1**	**2**	**3**	**4**	**5**	**1**	**2**	**3**	**4**	**5**	**1**	**2**	**3**	**4**
**beta-catenin**	12773922	13195308	9154429	14598402	24038742	12260052	10853839	8428637	12862500	7579068	4039068	6229764	2734448	3590748	10944024	3044027	5210525
**p-GSK3beta**	4003807	5894507	6136487	4969914	4508006	4055977	5488913	4432711	4076956	3698076	4142446	6092790	3721571	2148960	2999997	4779142	6370771
**GSK3beta 1**	3463902	5253066	4689204	3200613	6735690	5839392	7910994	8567949	6436326	12381291	4829877	12097800	8651253	3125577	10960560	7778316	8665527
**GSK3beta 2**	2491203	3839433	3273621	3322839	4580238	3955731	3474354	3892473	4162470	6801756	3382197	5865834	5888220	4006119	7917468	6614400	5493852
**GSK3beta (SUM)**	5955105	9092499	7962825	6523452	11315928	9795123	11385348	12460422	10598796	19183047	8212074	17963634	14539473	7131696	18878028	14392716	14159379
**CXCR6**	7664184	6230994	3345216	7812000	9479484	5556589	4718700	6279546	7760475	12381096	6669773	11345760	6601434	9310686	9936082	6075006	6521250
**GAPDH**	7998084	5967858	8167740	3352815	7629732	6226164	9582255	12164900	7762568	10809154	14886438	13730376	8953970	12356428	13284450	11666214	15037990

SET 2 (Raw Densitometry)																	
	CTRL			SORA					SBI					COMBO			
**protein**	**1**	**2**	**3**	**1**	**2**	**3**	**4**	**5**	**1**	**2**	**3**	**4**	**5**	**1**	**2**	**3**	**4**
**beta-catenin**	5056309	5362817	7115544	3957409	13775470	9136114	8215480	8095933	12848620	7164421	5333032	9463601	3643316	5945863	11627435	8440070	9375356
**p-GSK3beta**	1564232	1174808	6136487	1854628	1547360	1299220	5488913	4432711	1468586	1670100	4142446	6092790	3721571	1358462	1984626	4779142	6370771
**GSK3beta 1**	5178412	5048718	5153028	5423208	8812998	6935456	5023448	8029970	7685272	13029782	7003286	11644454	3603084	6100672	10781702	2598554	8794606
**GSK3beta 2**	2304852	2927900	3135038	4004212	3896634	2645256	2855168	3178282	4029824	4771850	3794262	5398204	2204874	3473428	3784496	1344440	2862806
**GSK3beta**	7483264	7976618	8288066	9427420	12709632	9580712	7878616	11208252	11715096	17801632	10797548	17042658	5807958	9574100	14566198	3942994	11657412
**CXCR6**	10512900	6700044	5675787	7118943	10364718	7962279	8139768	7636005	7947849	10091874	6005688	9838218	7344675	7216053	9648592	9281454	7760798
**GAPDH**	7861089	8570364	7271616	8073506	9949050	7730669	6844662	6362325	7982910	11925675	9717441	11036925	8679780	11554884	10568116	10652532	9919312

SET 1 (NORMALIZED to GAPDH)																	
	CTRL			SORA					SBI					COMBO			
**protein**	**1**	**2**	**3**	**1**	**2**	**3**	**4**	**5**	**1**	**2**	**3**	**4**	**5**	**1**	**2**	**3**	**4**
**beta-catenin**	1.597123	2.211063	1.120803	4.354073	3.150667	1.969118	1.132702	0.692865	1.65699	0.701171	0.271325	0.453721	0.305389	0.290598	0.823822	0.260927	0.346491
**p-GSK3beta**	0.504426	0.987709	0.751308	1.482311	0.590847	0.651441	0.572821	0.364385	0.525207	0.342124	0.27827	0.443745	0.415634	0.173914	0.225828	0.409657	0.423645
**GSK3beta**	0.744566	1.523578	0.974912	1.945664	1.483136	1.57322	1.18817	1.024293	1.365372	1.774704	0.551648	1.308313	1.623802	0.577165	1.421062	1.233709	0.941574
**CXCR6**	0.965585	1.044092	0.409564	2.329982	1.24244	0.892458	0.492441	0.516202	0.99973	1.145427	0.448044	0.826326	0.737263	0.75351	0.747948	0.520735	0.433652

SET 2 (NORMALIZED to GAPDH)																	
	CTRL			SORA					SBI					COMBO			
**protein**	**1**	**2**	**3**	**1**	**2**	**3**	**4**	**5**	**1**	**2**	**3**	**4**	**5**	**1**	**2**	**3**	**4**
**beta-catenin**	0.643207	0.62574	0.978537	0.490172	1.384602	1.181801	1.200275	1.27248	1.609516	0.600756	0.54881	0.857449	0.419748	0.514576	1.100237	0.792306	0.945162
**p-GSK3beta**	0.198984	0.137078	0.843896	0.229718	0.155528	0.189969	0.801926	0.696712	0.183966	0.140042	0.42629	0.552037	0.428763	0.117566	0.187794	0.448639	0.642259
**GSK3beta**	0.951937	0.930721	1.139783	1.167698	1.277472	1.239312	1.15106	1.76166	1.467522	1.492715	1.111151	1.544149	0.669137	0.828576	1.378315	0.370146	1.175224
**CXCR6**	1.337334	0.781769	0.78054	0.881766	1.04178	1.028093	1.189214	1.200191	0.995608	0.846231	0.618032	0.891391	0.846182	0.624502	0.912991	0.871291	0.782393

AVERAGE NORMALIZED (SET 1 & 2)																		
	CTRL			SORA					SBI					COMBO				
protein	1	2	3	1	2	3	4	5	1	2	3	4	5	1	2	3	4
beta-catenin	1.120165	1.418401	1.04967	2.422123	2.267634	1.57546	1.166489	0.982673	1.633253	0.650964	0.410068	0.655585	0.362568	0.402587	0.96203	0.526617	0.645826
p-GSK3beta	0.351705	0.562393	0.797602	0.856014	0.373188	0.420705	0.687373	0.530549	0.354587	0.241083	0.35228	0.497891	0.422198	0.14574	0.206811	0.429148	0.532952
GSK3beta	0.848252	1.22715	1.057347	1.556681	1.380304	1.406266	1.169615	1.392976	1.416447	1.633709	0.8314	1.426231	1.146469	0.70287	1.399689	0.801928	1.058399
CXCR6	1.151459	0.912931	0.595052	1.605874	1.14211	0.960276	0.840828	0.858196	0.997669	0.995829	0.533038	0.858858	0.791723	0.689006	0.83047	0.696013	0.608022

*Each column/number represents a mouse within that group.

*Each set includes two western blots that include all samples from tumors across groups.

*Both blots for each set were quantified for the same exposure time to ensure fair comparison across samples.

*Two technical replicates were done for all samples (Set 1 & 2) and points are average for both sets per mouse.

*****Values from "AVERAGE NORMALIZED (SET 1 & 2)" Table were used for graphs*****

<u>Figure S2:</u> SBI-457 attenuates sorafenib-induced nuclear accumulation of β-catenin in SK-Hep-1 cells

<u>Figure S2A: whole cell</u>

*fold change from DMSO values are graphed, calculated from B-catenin/GAPDH ratio of raw values

WB: anti-β-catenin

1)

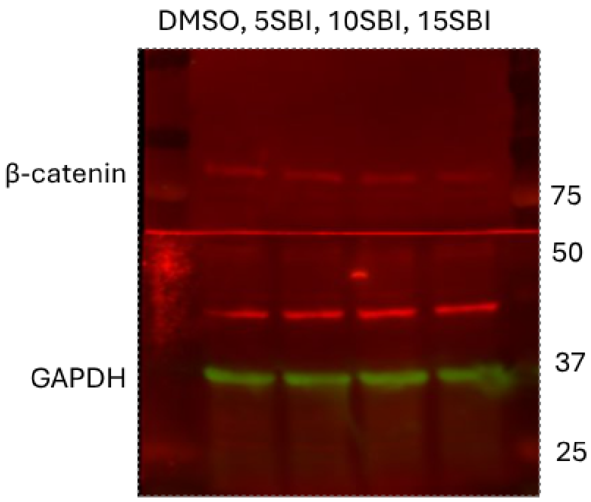

B-catenin		3750	3320	2870	1850
GAPDH		24100	20700	25100	24300
Ratio:					
B-catenin	#DIV/0!	0.155602	0.160386	0.114343	0.076132

fold change from WT DMSO	DMSO	5uM SBI-4	10uM SBI-	15uM SBI-
B-catenin	1	1.03075	0.734842	0.489273
CXCR6	1	1.492269	1.393218	1.235215

2)

DMSO, 5SBI, 10SBI, 15SBI   DMSO, 5SBI, 10SBI, 15SBI

β-catenin

GAPDH

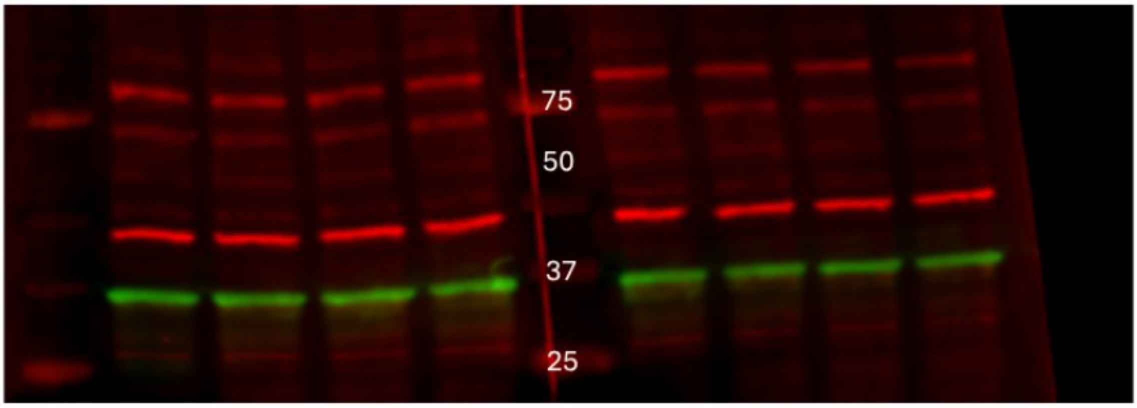

^Technical replicates, graphed average values

	lane 1	lane 2	lane 3	lane 4	lane 5	lane 6	lane 7	lane 8	lane 9	lane 10	lane 11	lane 12
B-catenin		10100	7800	7160	6490		7230	5100	4540	3720		
GAPDH		49900	48800	46300	42800		30100	27900	31000	29200		
Ratio:												
B-catenin	#DIV/0!	0.202405	0.159836	0.154644	0.151636	#DIV/0!	0.240199	0.182796	0.146452	0.127397	#DIV/0!	#DIV/0!

fold change from WT DMSO	DMSO	5uM SBI-4	10uM SBI-	15uM SBI-457	DMSO	5uM SBI-4	10uM SBI-	15uM SBI-457	DMSO	5uM SBI-4	10uM SBI-	15uM SBI-457
B-catenin	1	0.789685	0.764031	0.74917	1	0.761017	0.609709	0.530381	1	0.775351	0.68687	0.639775

3)

DMSO, 2.5sora, 5sora, 7.5sora, 10sora   DMSO, 2.5sora, 5sora, 7.5sora, 10sora

β-catenin

GAPDH

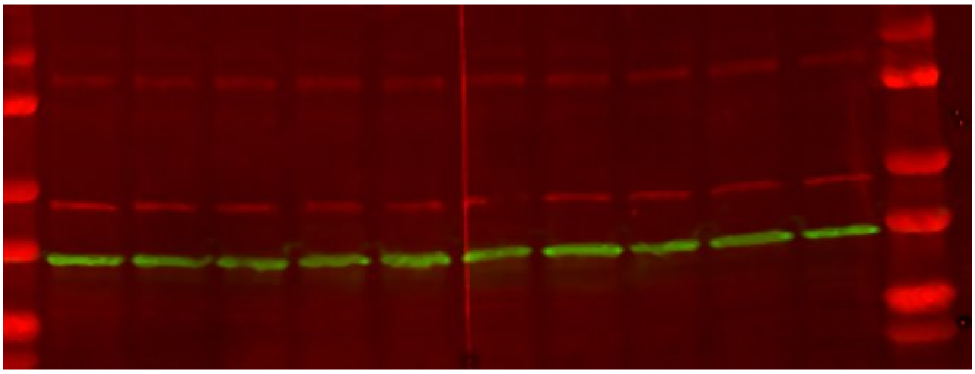

75
50
37
25

^Technical replicates, graphed average values

	lane 1	lane 2	lane 3	lane 4	lane 5	lane 6	lane 7	lane 8	lane 9	lane 10	lane 11
B-catenin		1060	887	1180	1460	1120	1090	1120	1160	1450	1100
GAPDH		43500	33100	29000	30100	44700	35400	48800	30700	39000	38700
Ratio:											
B-catenin	#DIV/0!	0.024368	0.026798	0.04069	0.048505	0.025056	0.030791	0.022951	0.037785	0.037179	0.028424

	first set					technical repeats					average of the two				
fold change from WT DMSO	DMSO	2.5uM SFE	5uM SFB	7.5uM SFE	10uM SFB	DMSO	2.5uM SFE	5uM SFB	7.5uM SFE	10uM SFB	DMSO	2.5uM SFE	5uM SFB	7.5uM SFE	10uM SFI
B-catenin	1	1.099712	1.669811	1.990535	1.028239	1	0.745375	1.227146	1.207481	0.923121	1	0.922544	1.448479	1.599008	0.9756[illegible]

4)

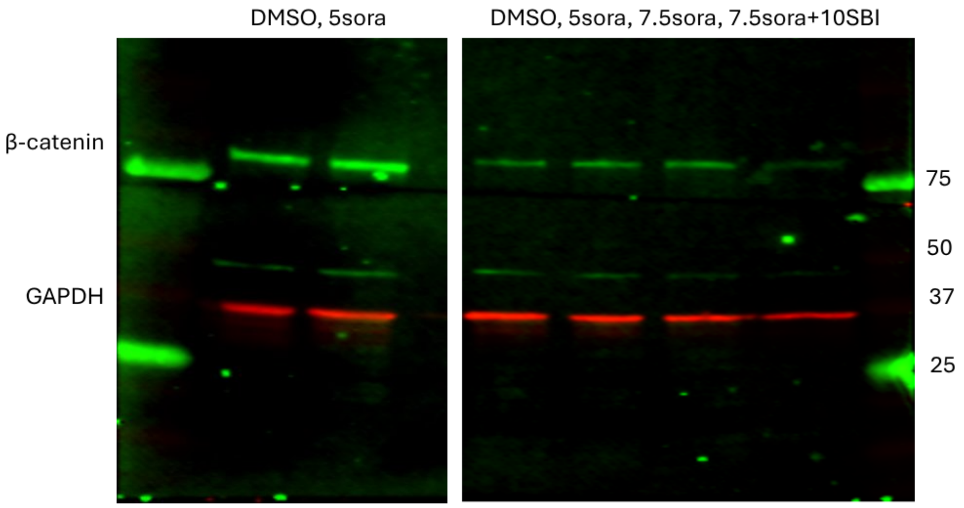

^Technical replicates,
graphed average values

B-catenin		387	482	106	148	144	71.4
GAPDH		36600	43100	50000	38200	35200	27600
Ratio:							
B-catenin	#DIV/0!	0.010574	0.011183	0.00212	0.003874	0.0040909	0.002587

								average of two			
fold change from SR DMSO	DMSO	5uM SFB		DMSO	5uM SFB	7.5uM SFB	7.5 + 10	DMSO	5uM SFB	7.5uM SFB	7.5 + 10
B-catenin	1	1.057645	#DIV/0!	1	1.827521	1.9296741	1.220263	1	1.442583	1.9296741	1.220263

5)

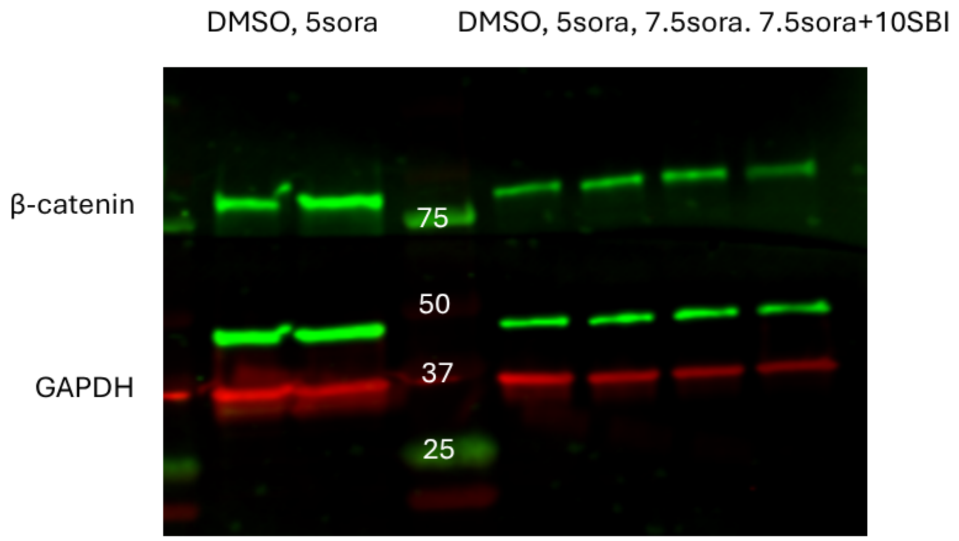

^Technical replicates,
graphed average values

B-catenin	1420	2970		741	957	965	636
GAPDH	28900	31100		36100	26500	23000	21800
Ratio:							
B-catenin	0.0491349	0.0954984	#DIV/0!	0.020526	0.036113	0.0419565	0.029174

								average of the two
DMSO	5uM SFB		DMSO	5uM SFB	7.5uM SFE	7.5 + 10		5uM SFB
1	1.943594		1	1.759361	2.044036	1.4213126		1.851478
1	1.0867293		1	1.270893	1.569565	1.2823618		1.178811

6)

DMSO, DMSO, 3SBI, 7.5sora, 7.5sora+3SBI, +5SBI, + 7.5SBI

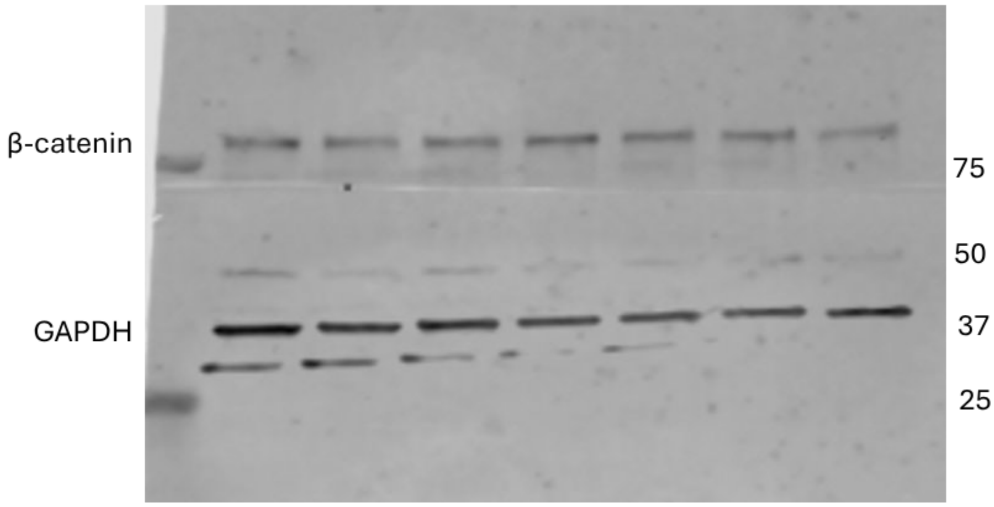

	lane 1	lane 2	lane 3	lane 4	lane 5	lane 6	lane 7	lane 8
B-catenin		325	212	274	317	243	259	166
GAPDH		38600	27700	29200	24300	21600	18600	29500
Ratio:								
B-catenin	#DIV/0!	0.00842	0.007653	0.009384	0.013045	0.01125	0.013925	0.005627

fold change from DMSO	DMSO	DMSO	3uM SBI-4	7.5uM sor	7.5uM sor	7.5uM sor	7.5uM sorafenib + 7.[illegible]	
B-catenin	1	1	1.167609	1.62324	1.399853	1.732673	0.70019	#DIV/0!

7)

DMSO, 3SBI, 7.5SBI, 7.5sora+3SBI DMSO, 7.5sora, 7.5sora+3SBI, +5SBI

β-catenin

GAPDH

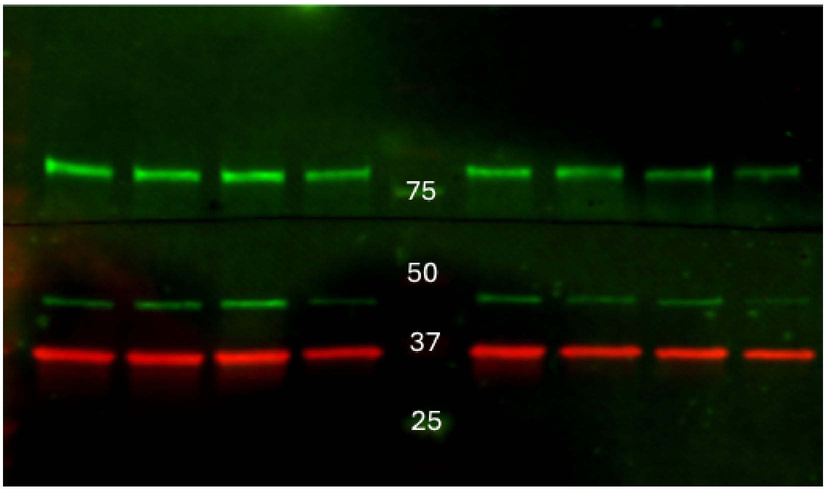

^Technical replicates, graphed average values

	lane 1	lane 2	lane 3	lane 4	lane 5	lane 6	lane 7	lane 8	lane 9	lane 10
B-catenin		364	356	424	214		280	394	380	266
GAPDH		28100	28400	33400	23700		34300	26600	25500	20300
Ratio:										
B-catenin	#DIV/0!	0.012954	0.012535211	0.012694611	0.009029536	#DIV/0!	0.008163	0.014812	0.014902	0.013103

fold change from DMSO	DMSO	SBI-457 3u	SBI-457 7.5uN	SFB + 3uM SBI-457		DMSO	sorafenib	SFB + 3uM S	SFB + 5uM	average<br>SFB + 3uM SBI-457
B-catenin	1	0.967691	0.979996052	0.697060324	#DIV/0!	1	1.814474	1.8254902	1.605172	1.261275

8)

DMSO, 3SBI, 5sora

β-catenin

GAPDH

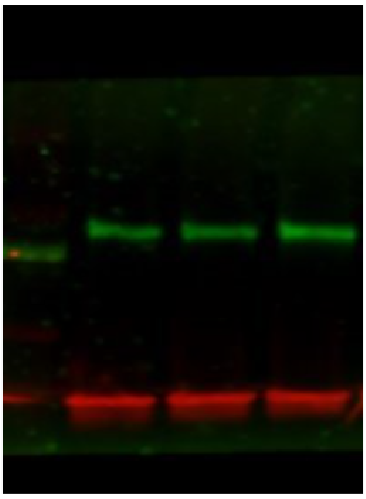

75
50
37
25

B-catenin		684	633	916
GAPDH	6120	43700	46100	45200
Ratio:				
B-catenin	0	0.015652	0.013731	0.020265

fold change from DMSO	DMSO	3uM SBI-457	5uM sorafenib
B-catenin	1	0.87726	1.294739

<u>Figure S2B&C: nuclear and cytoplasmic fractions</u>

*fold change from DMSO values (for each fraction) are graphed, calculated from B-catenin/loading control ratio of raw values

WB: anti-β-catenin

1)

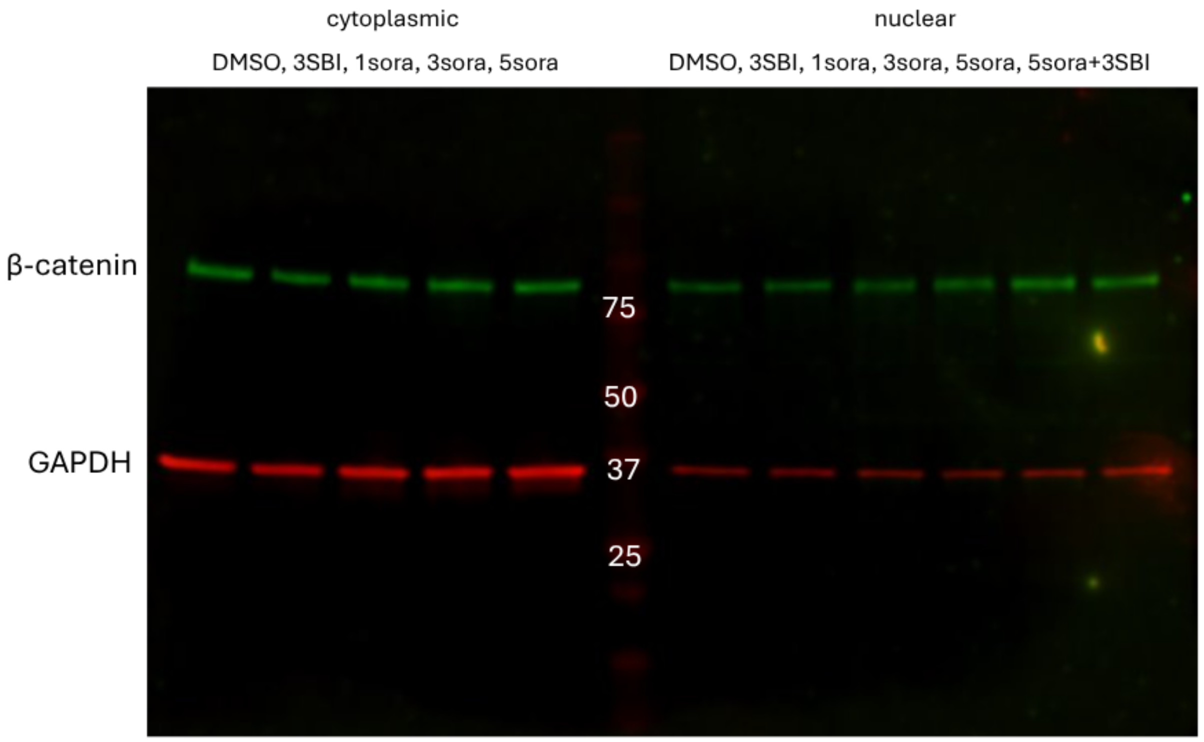

B-catenin	1640	1110	1220	1040	1030		511	530	505	654	110	1350
GAPDH	19100	15100	15800	13400	18500		6420	6290	5280	4910	5290	9500
Ratio:												
B-catenin	0.085864	0.07351	0.077215	0.077612	0.055676	#DIV/0!	0.079595	0.084261	0.095644	0.133198	0.020794	0.142105
fold change from DMSO	DMSO	3uM SBI-4	1uM SFB	3uM SFB	5uM SFB	marker	DMSO	3uM SBI-4	1uM SFB	3uM SFB	5uM SFB	5uM sorafenib + 3uM 457
B-catenin	1	0.856122	0.899274	0.903895	0.648418		1	1.058618	1.201632	1.673441	0.261247	1.785354

2)

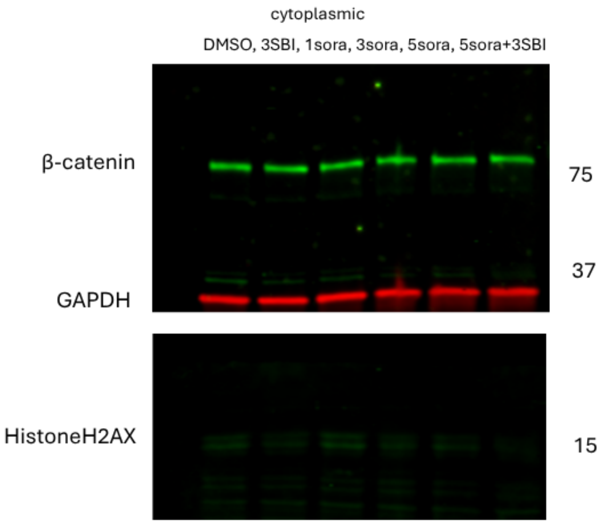

B-catenin	1390	1510	1490	1420	1480	1350
GAPDH/ Histone	43600	45100	51400	41000	42700	42900
Ratio:						
B-catenin	0.031881	0.033481	0.028988	0.034634	0.03466	0.031469

	cytoplasmic					
fold change from DMSO	DMSO	3uM SBI-	1uM SFB	3uM SFB	5uM SFB	5uM soraf
B-catenin	1	1.0502	0.909274	1.086366	1.08719	0.98707

3)

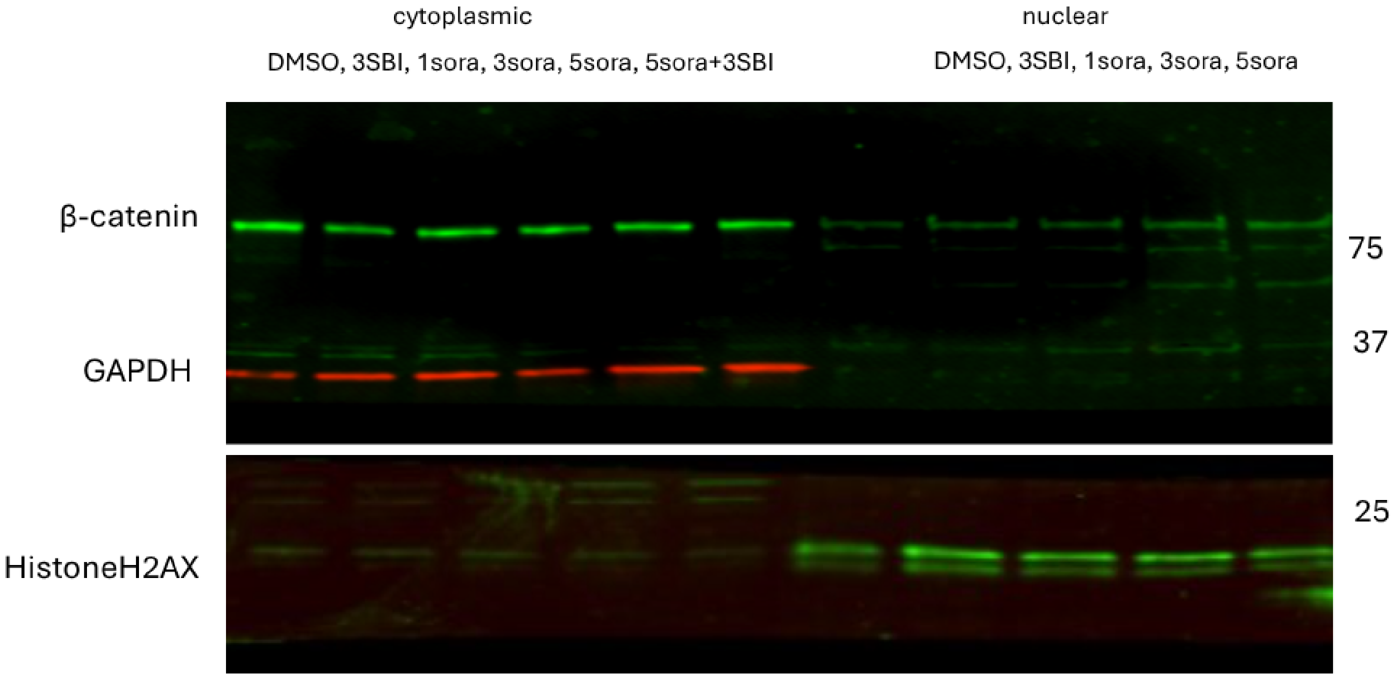

B-catenin	1290	677	1100	844	1010	1020	246	372	289	358	358
GAPDH/histone	25900	39700	47200	26400	59200	59400	403	850	628	579	213
Ratio:											
B-catenin	0.04981	0.0170529	0.0233051	0.03197	0.0170608	0.0171717	0.61042	0.4376471	0.4601911	0.61831	1.6807512

	cytoplasmic						nuclear				
fold change from DMSO	DMSO	3uM SBI-4	1uM sorafe	3uM sora	5uM sorafe	5uM sorafe	DMSO	3uM SBI-4	1uM sorafe	3uM sora	5uM sorafe
B-catenin	1	0.3423799	0.4679083	0.64187	0.3425388	0.3447655	1	0.7169584	0.7538903	1.01292	2.7534257

4)

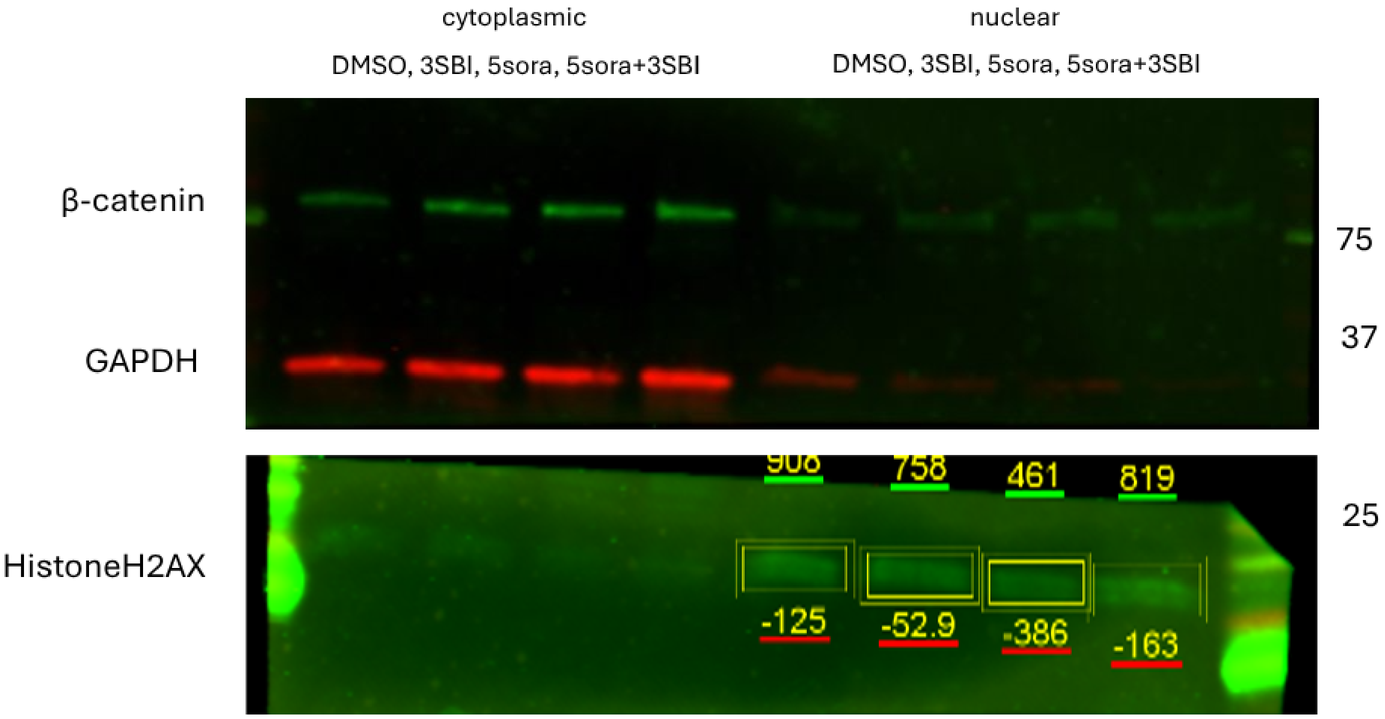

B-catenin		386	582	667	688	177	211	204	193
GAPDH/HISTONE		43000	52000	50500	63400	908	758	461	819
Ratio:									
B-catenin	#DIV/0!	0.008977	0.011192	0.013208	0.010852	0.194934	0.278364	0.442516	0.235653

	cytoplasm				nuclear			
fold change from each DMSO	DMSO	3uM SBI-457	5uM sorafenib	5uM sorafenib + 3uM SBI-457	DMSO	3uM SBI-457	5uM sorafenib	5uM sorafenib + 3uM SBI-457
B-catenin	1	1.246811	1.471349	1.208872	1	1.427992	2.270083	1.208888

5)

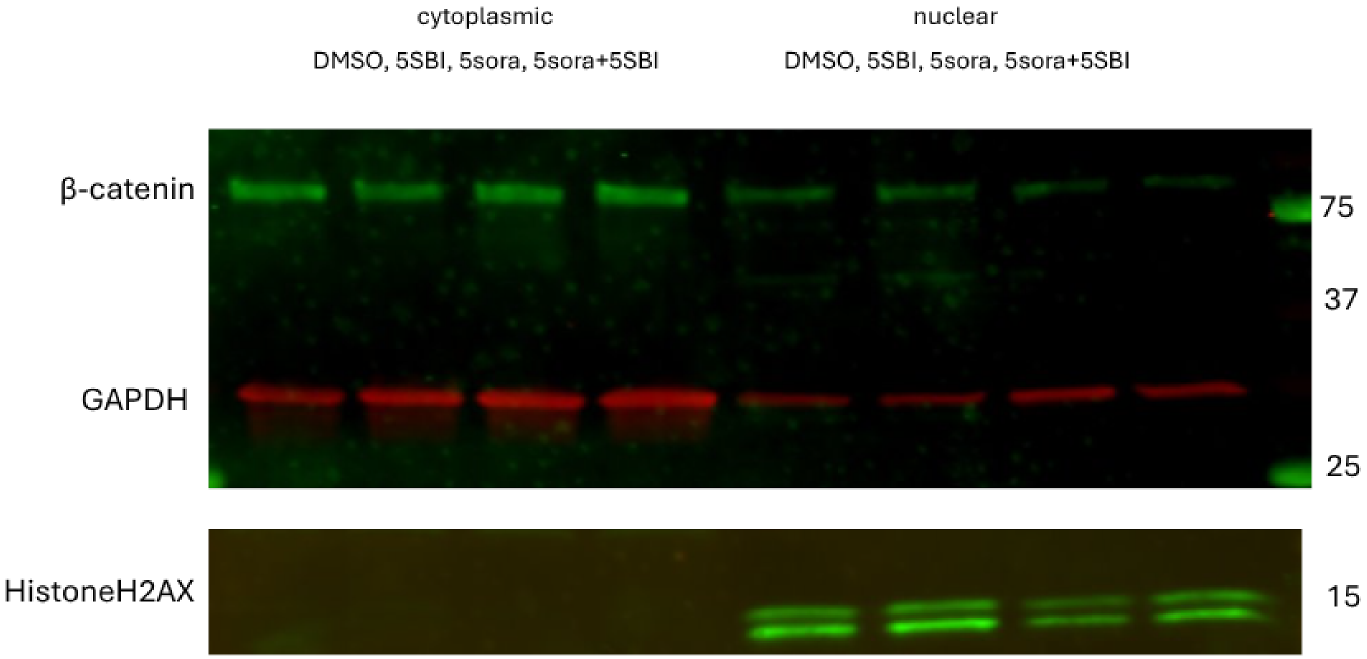

B-catenin		813	509	779	812	328	297	279	309
GAPDH/Histone H2A.X		39100	39700	51500	52100	6720	7570	3100	5470
Ratio:									
	#DIV/0!	0.020793	0.012821	0.015126	0.015585	0.04881	0.039234	0.09	0.05649

	cytoplasm				nuclear			
fold change from each DMSO	DMSO	5uM SBI-457	5uM sorafenib	5uM sorafenib + 5uM SBI-457	DMSO	5uM SBI-457	5uM sorafenib	5uM sorafenib + 5uM SBI-457
B-catenin	1	0.616614	0.727472	0.749557	1	0.803815	1.843902	1.157355

6)

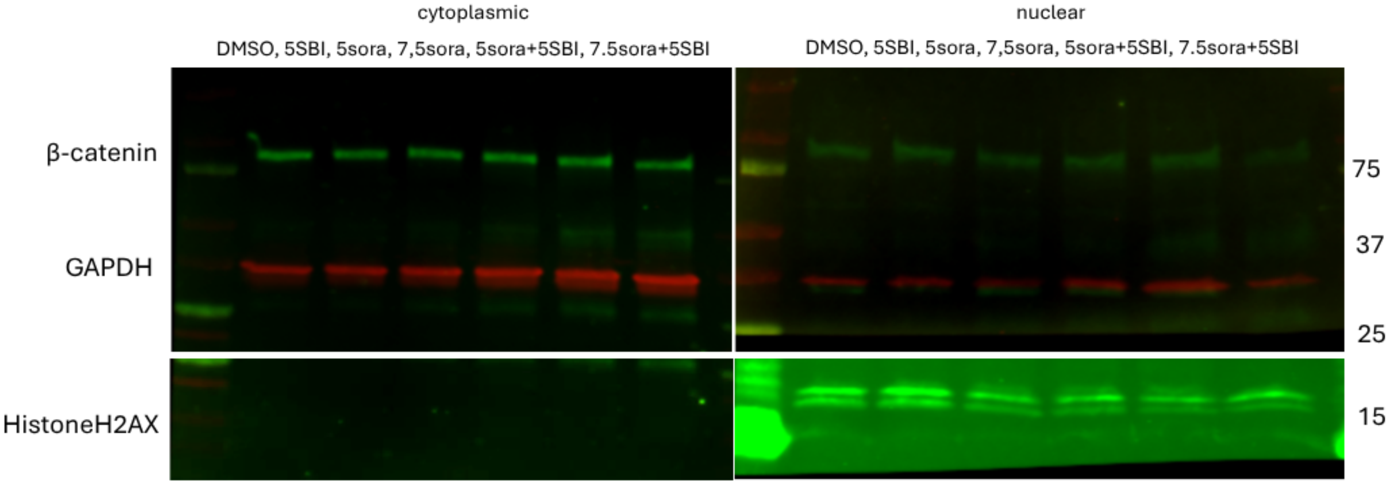

B-catenin		1580	1340	1420	1720	1800	1780	352	399	301	378	461	185
GAPDH/Histone H2A.X		44200	41300	44100	48200	55900	58700	2350	2940	1510	1450	1160	1590
Ratio:													
	#DIV/0!	0.035747	0.032446	0.0322	0.035685	0.0322	0.030324	0.149787	0.135714	0.199338	0.26069	0.397414	0.116352

	cytoplasm (48h)						nuclear (48h)					
fold change from each DMSO	DMSO	5uM SBI-457	5uM sorafenib	7.5uM sorafenib	5uM sorafenib + 5uM SBI-457	7.5uM sorafenib + 5uM SBI-457	DMSO	5uM SBI-457	5uM sorafenib	7.5uM sorafenib	5uM sorafenib + 5uM SBI-457	7.5uM sorafenib + 5uM SBI-457
B-catenin	1	0.907653	0.900772	0.998267	0.900795	0.848295	1	0.906047	1.330806	1.7404	2.653189	0.776783

7)

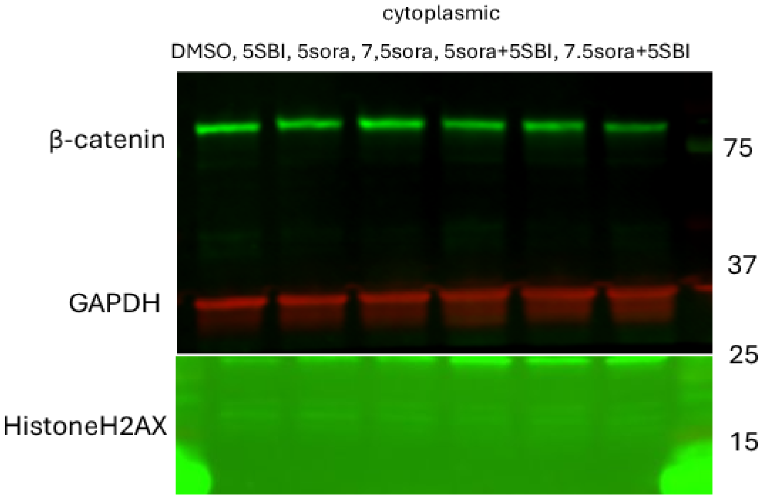

B-catenin		2590	220	2780	1910	1830	1150
GAPDH/Histone H2A.X		53700	48400	38800	38400	43200	44800
Ratio:							
	#DIV/0!	0.048231	0.004545	0.071649	0.04974	0.042361	0.02567

fold change from each DMSO	DMSO	5uM SBI-457	5uM sorafenib	7.5uM sorafenib	5uM sorafenib + 5uM SBI-457	7.5uM sorafenib + 5uM SBI-457
B-catenin	1	0.094244	1.485551	1.03128	0.878298	0.532224

8)

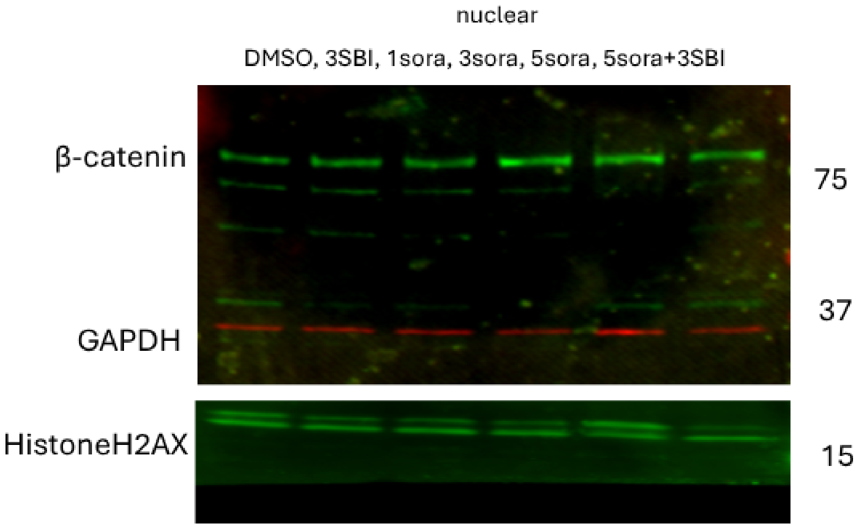

B-catenin	129	151	139	201	179	125
GAPDH/Histone	2260	1580	1810	1660	1440	1550
Ratio:						
B-catenin	0.05708	0.09557	0.076796	0.121084	0.124306	0.080645

fold change from DMSO	DMSO	3uM SBI-4	1uM soraf	3uM soraf	5uM soraf	5uM soraf
B-catenin	1	1.67432	1.345411	2.121322	2.177756	1.412853

Figure S3: Correlation of treatment related cell death in HCC cells to CXCR6, soluble CXCL16 and β-catenin expression

Figure S3A: total β-catenin in whole cell lysates

WB: anti-β-catenin

1)

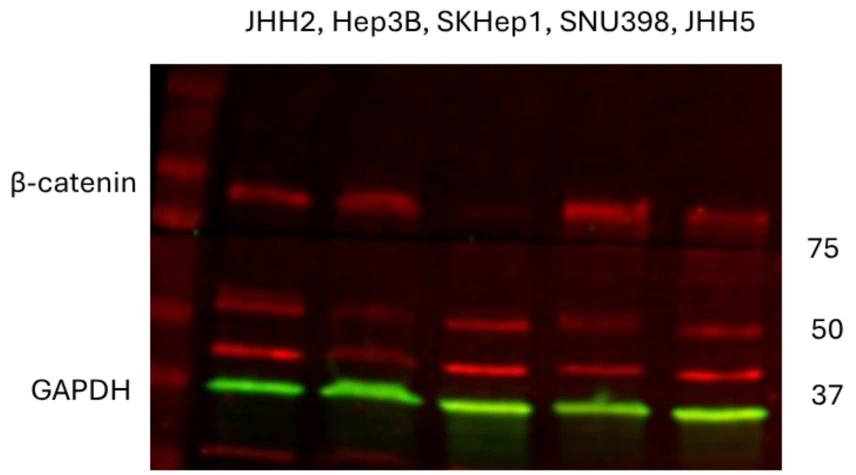

total B-catenin		5550	5860	957	9300	4780
GAPDH/Beta actin		31200	42200	41200	33900	41700
Ratio:						
total β-catenin	#DIV/0!	0.177884615	0.138862559	0.023228155	0.274336283	0.114628297

2)

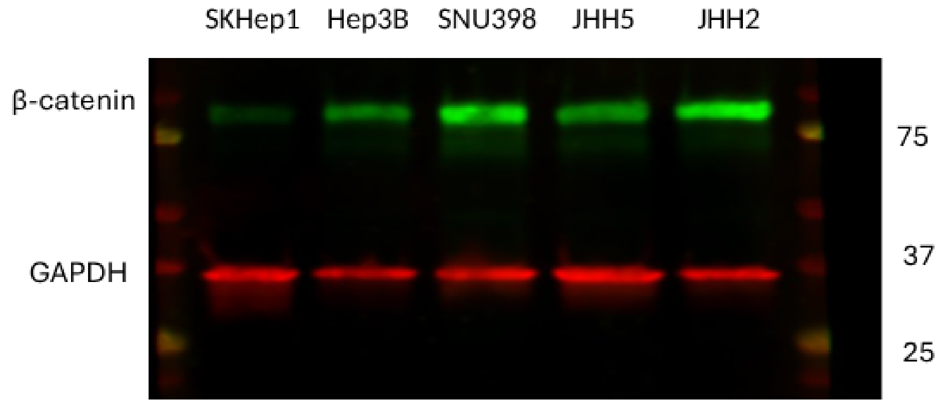

**total B-catenin**	1320	3770	8730	5270	8760
**GAPDH**	26000	20000	24600	34100	21400
Ratio:					
**total B-catenin**	0.050769	0.1885	0.354878	0.154545	0.409346

3)

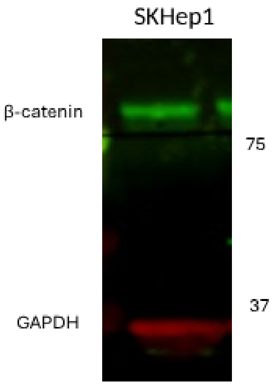

total B-catenin		1130
GAPDH		20000
Ratio:		
_	#DIV/0!	0.0565

4)

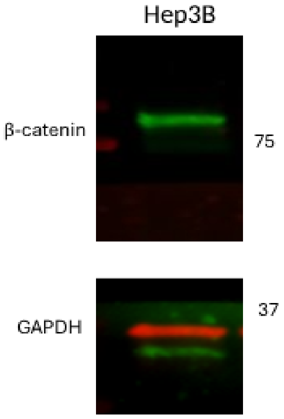

total beta catenin		9230
GAPDH		44100
Ratio:		
total beta catenin	#DIV/0!	0.209297

5)

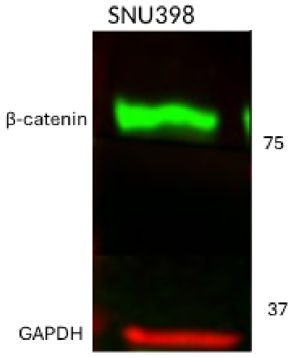

total B-catenin		16400
GAPDH		17300
Ratio:		
total B-catenin	#DIV/0!	0.947977

6)

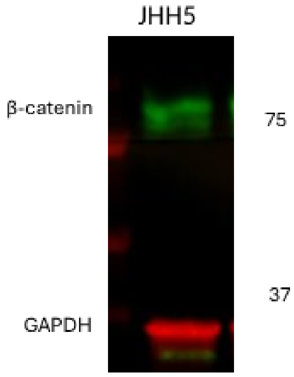

total B-catenin		3920
GAPDH		22000
Ratio:		
total B-catenin	#DIV/0!	0.178182

Figure S4C: total CXCR6 in whole cell lysates

WB: anti-CXCR6

1)

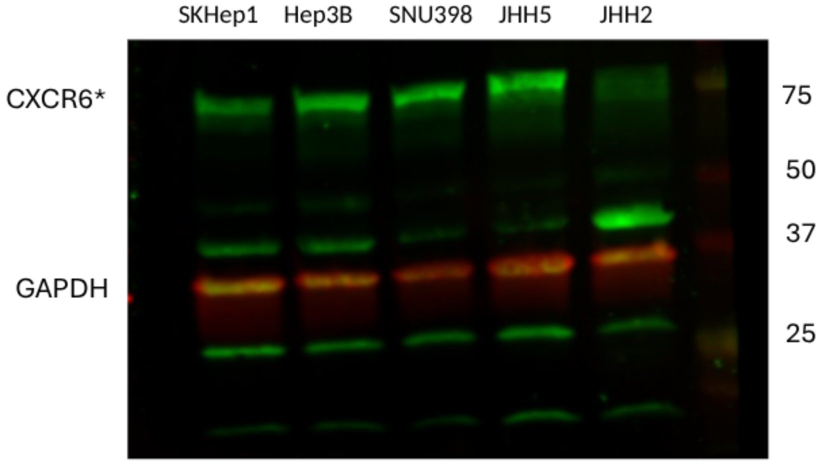

*All Alexa Fluor 488

**CXCR6 high MW band**	5220	7120	6810	9800	4770
normalized signal	#DIV/0!	#DIV/0!	#DIV/0!	#DIV/0!	#DIV/0!
**CXCR6 (39-44kDa)**	2600	2470	872	1090	7890
normalized signal	#DIV/0!	#DIV/0!	#DIV/0!	#DIV/0!	#DIV/0!
**GAPDH**	43900	33800	25300	46700	44800
Ratio:					
**CXCR6 high MW band**	0.118907	0.210651	0.26917	0.20985	0.106473
**CXCR6 (39-44kDa)**	0.059226	0.073077	0.034466	0.02334	0.176116

2)

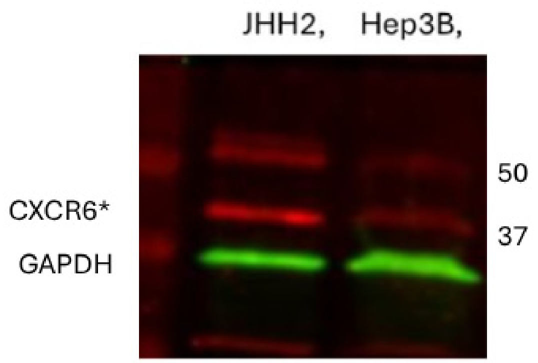

*All Alexa Fluor 680

CXCR6		4480	1790
GAPDH/Beta actin		31200	42200
Ratio:			
CXCR6	#DIV/0!	0.143589744	0.042417062

3)

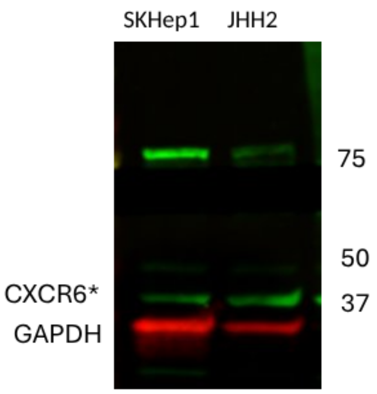

*All Alexa Fluor 488

CXCR6	3100	5320
High MW band	9220	2330
GAPDH/beta actin	55800	25600
Ratio:		
CXCR6	0.055556	0.207813
High MW CXCR6	0.16523	0.09101

4)

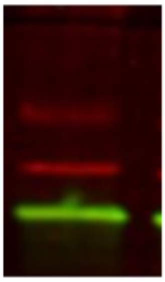

*All Alexa Fluor 680

CXCR6		3060
GAPDH/Beta actin		33900
Ratio:		
CXCR6	#DIV/0!	0.090265487

5)

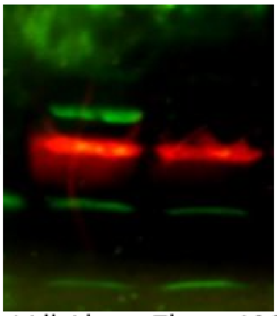

*All Alexa Fluor 488

CXCR6	881	79.7
GAPDH	32600	17600
Ratio:		
CXCR6	0.027025	0.004528

6)

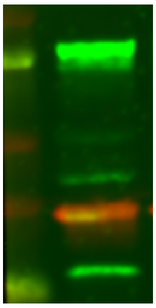

*All Alexa Fluor 488

**CXCR6 high MW**	7890
**CXCR6**	729
**GAPDH**	25600
Ratio:	
**CXCR6 high MW**	0.308203
**CXCR6**	0.028477
